# Metabolic Engineering of Terpenoid Biosynthesis in Medicinal Plants: From Genomic Insights to Biotechnological Applications

**DOI:** 10.3390/cimb47090723

**Published:** 2025-09-05

**Authors:** Changfeng Guo, Si Xu, Xiaoyun Guo

**Affiliations:** 1 National Center for Traditional Chinese Medicine Inheritance and Innovation, Guangxi Botanical Garden of Medicinal Plants, Nanning 530012, China; cfguo2020@163.com; 2 School of Life Science and Bioengineering, Jining University, Qufu 273155, China; xusi@jnxy.edu.cn

**Keywords:** metabolic engineering, CRISPR-Cas9, multi-omics integration, heterologous expression, synthetic biology, artemisinin, paclitaxel, scale-up challenges

## Abstract

Terpenoids, which are essential pharmaceutical compounds, encounter significant production challenges due to their low yields in native plants and associated ecological concerns. This review summarizes recent advances in metabolic engineering strategies applied across three complementary platforms: native medicinal plants, microbial systems, and heterologous plant hosts. We present how the “Genomic Insights to Biotechnological Applications” paradigm, supported by multi-omics technologies such as genomics, transcriptomics, metabolomics, and related disciplines, contributes to advancing research in this field. These technologies enable the systematic identification of key biosynthetic genes and regulatory networks. CRISPR-based tools, enzyme engineering, and subcellular targeting are presented as pivotal transformative strategies in advancing metabolic engineering approaches. Strategic co-expression and optimization approaches have achieved substantial improvements in product yields, as demonstrated by a 25-fold increase in paclitaxel production and a 38% enhancement in artemisinin yield. Persistent challenges, such as metabolic flux balancing, cytotoxicity, and scale-up economics, are discussed in conjunction with emerging solutions, including machine learning and photoautotrophic chassis systems. We conclude by proposing a strategic roadmap for industrial translation that highlights the essential integration of systems biology and synthetic biology approaches to accelerate the transition of terpenoid biomanufacturing from discovery to commercial-scale application.

## 1. Introduction

Terpenoids, a diverse and structurally complex class of natural products, serve as foundational components for numerous life-saving pharmaceuticals, thereby making a significant impact on global healthcare. Traditional sourcing of pharmaceutical terpenoids from native plants is confronted with a combination of significant challenges. Unsustainable yields are frequently below 0.05% dry weight. Growth cycles are prolonged. Some species, such as *Panax ginseng*, require 5 to 7 years to mature. Environmental variability results in inconsistent biochemical profiles. Ecological degradation caused by over-harvesting further compounds these issues [1,2]. Metabolic engineering provides a sustainable solution through enhanced production across three complementary platforms: (i) native medicinal plants, which enable optimized in planta biosynthesis while preserving the natural genetic context [3]; (ii) microbial chassis systems, such as bacteria and yeast, which provide rapid growth capabilities, well-established genetic toolkits, and scalable fermentation processes [4]; and (iii) heterologous plant hosts, such as *Nicotiana benthamiana*, which offer eukaryotic post-translational modification capabilities and advanced subcellular compartmentalization features [5,6].

A “Genomic Insights to Biotechnological Applications” paradigm serves as a driving force behind contemporary advancements in terpenoid production. Multi-omics technologies systematically elucidate biosynthetic pathways, establishing a robust foundation for targeted engineering. Genome mining has led to the identification of taxadiene synthase, which functions as the gateway enzyme for paclitaxel biosynthesis in *Taxus* species [7]. Transcriptomic analyses have revealed jasmonate-induced expression patterns of artemisinin biosynthetic pathway genes in *Artemisia annua*, thereby informing targeted strategies for pathway activation [8]. Heterologous expression, supported by integrated proteomic and metabolomic analyses, enables pathway reconstitution and functional validation of genes, as exemplified by the successful reconstruction of Panax ginseng ginsenoside biosynthesis pathways in yeast chassis systems [9]. Building upon genomic insights, next-generation toolkits facilitate platform-specific metabolic interventions. Studies have demonstrated that CRISPR-Cas9-mediated knockout of competing pathways in plants enhances terpenoid production, underscoring the efficacy of in planta metabolic rewiring [10].

Persistent challenges remain, including the difficulty of balancing metabolic flux in complex networks, the cytotoxic effects of oxidized terpenoid intermediates in microbial hosts, the limited availability of cytochrome P450 enzymes (which catalyze essential oxidation reactions, such as hydroxylations, that introduce functionality and structural diversity to terpenoid scaffolds), and limitations related to scale-up, such as the high production costs associated with plant cell culture. Future progress depends on three frontiers: (i) integration of systems biology, particularly genome-scale metabolic modeling for predictive pathway design [11]; (ii) development of photoautotrophic chassis systems aimed at reducing carbon dependency and enhancing sustainability [12]; and (iii) economically viable and sustainable bioprocessing platforms that enable commercial deployment [13]. This review is structured to first present a comparative analysis of the major production platforms for terpenoids (Section 2) and to provide a foundational understanding of terpene biosynthesis pathways and their regulatory mechanisms in medicinal plants (Section 3). Following this, we examine the core aspects of the topic, detailing the comprehensive range of metabolic engineering strategies applied across various host systems, ranging from enzyme overexpression to CRISPR-mediated genome editing (Section 4). We then discuss how advances in genomics and multi-omics technologies contribute critical insights that facilitate target identification and pathway elucidation (Section 5). Section 6 addresses the practical biotechnological applications of these strategies, including laboratory-scale studies and the challenges associated with scaling up production. In Section 7, we critically analyze the persistent challenges and bottlenecks that currently hinder further advancements in the field. Finally, Section 8 outlines perspectives on future opportunities and emerging directions, accompanied by a proposed strategic roadmap for the development of this area. Throughout the review, we emphasize the essential integration of systems biology and synthetic biology approaches to accelerate the progression of terpenoid biomanufacturing from discovery to commercial-scale implementation.

## 2. Comparative Analysis of Terpenoid Production Systems

The selection of an appropriate production platform is of critical importance for the successful and economically viable biomanufacturing of terpenoids. Each of the three primary platforms presents a unique combination of advantages and inherent limitations. These platforms include native medicinal plants, microbial chassis, and heterologous plant hosts. The optimal strategy is not universally applicable but is heavily influenced by the specific characteristics of the target terpenoid, the intended production scale, and the available technological and financial resources. To support informed decision-making, we present a concise, comparative overview of these platforms (Table 1), evaluating them based on key criteria such as maximum achieved yields, technological maturity, scalability, cost implications, and compatibility with different terpenoid classes.

In summary, the comparative analysis highlights that no single platform is universally superior for terpenoid production; each demonstrates strengths in specific applications. Microbial chassis, particularly *Saccharomyces cerevisiae*, currently represent the most advanced and scalable technology for the production of a broad spectrum of terpenoid precursors and simpler molecules, as demonstrated by the industrial-scale production of artemisinic acid [16]. Their rapid growth, well-characterized genetics, and advanced engineering tools render them particularly suitable for the production of molecules whose biosynthetic pathways can be fully reconstructed and optimized within a prokaryotic or fungal system.

Conversely, heterologous plant hosts such as *N. benthamiana* serve as an unparalleled eukaryotic testbed and production system for highly complex biosynthetic pathways that require plant-specific cytochrome P450s and glycosyltransferases. Their strength lies in enabling rapid prototyping and the production of small to medium quantities of high-value, structurally complex terpenoids that remain challenging to produce in microbial systems [21]. Finally, the engineering of native medicinal plants themselves remains a crucial strategy, particularly for compounds where the biosynthetic pathway has not been fully elucidated or where the complex cellular context and storage structures are difficult to reconstitute de novo [22]. This approach is most appropriate for achieving incremental yield improvements within established agricultural systems, as opposed to enabling de novo production of novel compounds [23]. Future progress will likely depend on intelligent, integrated strategies that capitalize on the distinct advantages of each platform. These strategies involve utilizing microbial systems for scalable precursor synthesis, plant-based systems for complex modifications, and synthetic biology tools to seamlessly integrate these processes [24,25].

## 3. Fundamentals of Terpene Biosynthesis in Medicinal Plants: Biosynthetic Pathways and Regulatory Mechanisms

### 3.1. Core Pathways: From Precursor Biosynthesis to Structural Diversification

**Figure 1:**
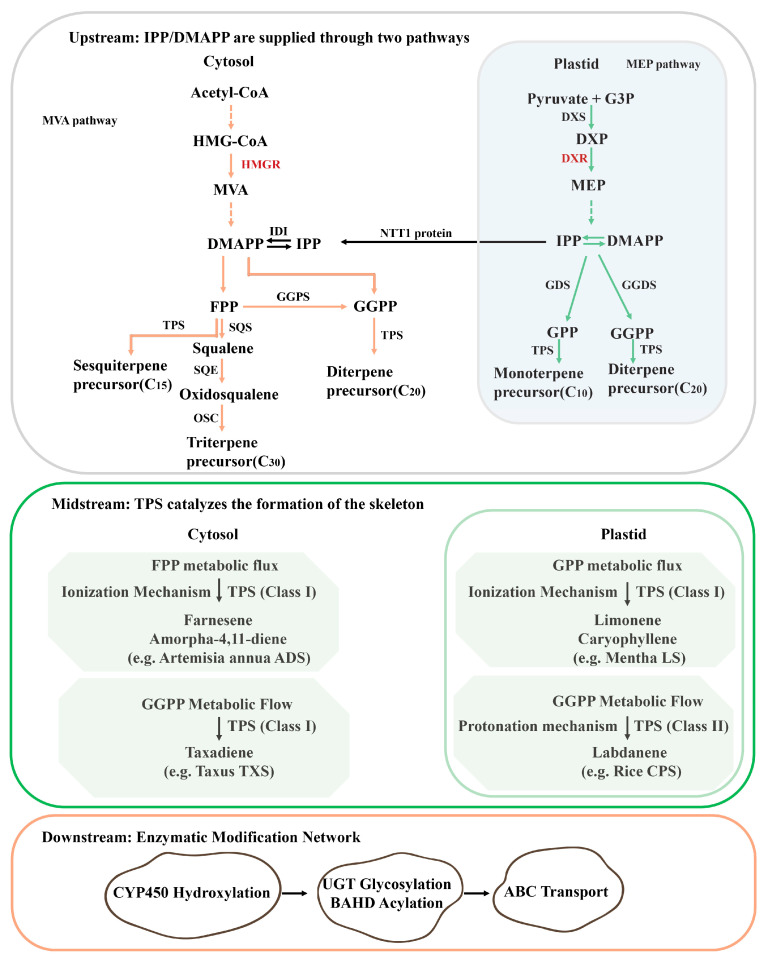
Metabolic pathways for terpenoid biosynthesis in medicinal plants. Upstream precursor supply: Isopentenyl diphosphate (IPP) and dimethylallyl diphosphate (DMAPP) are synthesized through the cytosolic mevalonate (MVA) pathway and plastidial methylerythritol phosphate (MEP) pathway. Key enzymes, highlighted in red, include 3-hydroxy-3-methylglutaryl-CoA reductase (HMGR) and 1-deoxy-D-xylulose-5-phosphate synthase (DXS). Midstream carbon skeleton formation: Terpene synthases (TPS) catalyze the cyclization of prenyl diphosphates (GPP, FPP, GGPP) into diverse terpenoid scaffolds, such as mono-, sesqui-, di-, and triterpenes. Downstream enzymatic modifications: Cytochrome P450s (CYPs), UDP-glycosyltransferases (UGTs), acyltransferases (BAHD), and ABC transporters mediate oxidation, glycosylation, acylation, and subcellular transport, thereby contributing to the formation of bioactive terpenoids.

Terpenoid biosynthesis begins with the formation of the universal C_5_ precursors, isopentenyl diphosphate (IPP) and dimethylallyl diphosphate (DMAPP), via two spatially distinct pathways in medicinal plants. From the perspective of metabolic engineering, the pathway is commonly classified into upstream, midstream, and downstream stages (Figure 1). The cytosolic mevalonate (MVA) pathway utilizes acetyl-CoA to generate farnesyl diphosphate (FPP, C_15_), which serves as the precursor for sesquiterpenes (C_15_) and triterpenes (C_30_). The pathway’s rate-limiting enzyme, 3-hydroxy-3-methylglutaryl-CoA reductase (HMGR), is subject to tight regulation by sterol-mediated feedback inhibition. Recent studies on metabolic engineering in *A. annua* demonstrate that targeted overexpression of the rate-limiting enzyme HMGR can enhance metabolic flux toward artemisinin biosynthesis. Overexpression of HMGR has been shown to specifically increase carbon allocation to sesquiterpene production. When the HMGR gene from *Catharanthus roseus* (CrHMGR) is introduced into *A. annua*, artemisinin yield increases by 22.5% to 38.9% compared to non-transgenic controls [26]. Concurrently, the plastidial methylerythritol phosphate (MEP) pathway catalyzes the condensation of pyruvate and glyceraldehyde-3-phosphate to form 1-deoxy-D-xylulose-5-phosphate (DXP), which is subsequently converted into isopentenyl diphosphate (IPP) and dimethylallyl diphosphate (DMAPP), providing precursors for monoterpenes (GPP, C_10_) and diterpenes (GGPP, C_20_). The enzyme responsible for the first committed and rate-limiting step in this pathway, 1-deoxy-D-xylulose-5-phosphate synthase (DXS), is therefore a key determinant of flux through the MEP pathway. This pathway is regulated by light, with DXS representing a central regulatory target. In *A. annua*, the blue-light receptor AaCRY1 phosphorylates and activates AaDXS, thereby enhancing the synthesis of artemisinin precursors [27].

The core objective of upstream metabolic engineering is to optimize the supply of the universal precursors, isopentenyl diphosphate (IPP) and dimethylallyl diphosphate (DMAPP), which serves as a foundational strategy for enhancing the overall flux of terpene biosynthesis. The biosynthesis of terpenoids in medicinal plants requires efficient exchange of precursors between organelles, a process that remains incompletely understood. A prime example is the hypothesized export of IPP from the plastidial MEP pathway to the cytosol, which is critical for supplying substrate for sesquiterpene and triterpene biosynthesis but is thought to occur via an as-yet-unidentified transporter [28]. Synthetic biology strategies can overcome the limitations imposed by cytosolic metabolic competition. Relocalization of monoterpene synthases to the chloroplasts of *N. tabacum*, by leveraging the high endogenous levels of the geranyl diphosphate (GPP) precursor, resulted in a 4.5-fold increase in monoterpene production compared to cytosolic localization [29]. Furthermore, reconstructing the entire cytosolic mevalonate (MVA) pathway within chloroplasts significantly increased triterpenoid yields through compartmentalized metabolic channeling [30]. Additionally, novel isopentenyl phosphate kinase (IPK) pathways provide simplified routes to isopentenyl diphosphate (IPP) and dimethylallyl diphosphate (DMAPP), requiring fewer enzymatic steps and utilizing only ATP as a cofactor [31]. Metabolic engineering strategies, such as pathway modularization and key enzyme optimization, have substantially improved microbial terpenoid production. Notably, a pivotal study has demonstrated that relocating the terpene biosynthesis pathway to the peroxisomes of engineered *S. cerevisiae* can enhance the production of specific monoterpene compounds up to 125-fold compared to cytoplasmic expression [32]. Similarly, an ERG20 enzyme fusion strategy, which involves constructing a bifunctional protein by fusing the native farnesyl diphosphate synthase (Erg20p) with a heterologous monoterpene synthase, resulted in a remarkable 340-fold increase in monoterpene yield. This approach improves production efficiency by enabling efficient substrate channeling of the geranyl diphosphate (GPP) intermediate, thereby reducing its diffusion and diversion into competing sterol biosynthetic pathways [33]. Furthermore, stepwise optimization of the endogenous mevalonate (MVA) pathway led to a 56-fold increase in total terpenoid titer [34,35].

Midstream metabolic engineering primarily aims to optimize terpene synthases (TPSs) to achieve efficient and highly specific catalysis of diverse carbon skeleton formations. Terpene synthases (TPSs) catalyze the cyclization of prenyl diphosphates (GPP, FPP, GGPP) into diverse carbon skeletons (Figure 1). The TPS gene family contributes to the structural diversity of terpenoids through functional diversification. The genome of *Salvia miltiorrhiza* is predicted to contain a large number of TPS members, among which 6–8 core enzymes (SmCPS1/2/4/5, SmKSL1, SmSTPS1–3) have been functionally verified [36,37]. Notably, SmKSL1b specifically catalyzes the formation of the tanshinone precursor, tanshinene [38]. Structure-guided engineering has improved catalytic specificity. The X-ray crystallographic structure of taxadiene synthase (TcTS) (resolution: 1.82 Å) reveals that its βγ domain regulates cyclization fidelity through a substrate confinement mechanism. Single-point mutations, such as I571T in *Gossypium arboreum* (+)-δ-cadinene synthase, can alter the product profile [39].

Downstream optimization strategies focus on the functional modification of carbon skeletons, including processes such as oxidation and glycosylation, as well as product transport. These steps collectively represent critical rate-limiting factors that determine the final product yield and structural diversity. Post-skeleton modifications enhance bioactivity and stability. Cytochrome P450s (CYPs) introduce hydroxyl and other functional groups, thereby significantly diversifying the chemical structures of plant secondary metabolites. In *Tripterygium wilfordii*, members of the CYP82D family of P450s have been shown to catalyze the key C14-hydroxylation reaction, which is an essential step in the biosynthetic pathway of triptolide, a potent anti-inflammatory compound [40]. In *A*. *annua*, the key enzyme AaCYP71AV1 catalyzes the conversion of amorpha-4,11-diene to artemisinic acid, a precursor of the antimalarial drug artemisinin [41]. Glycosyltransferases (UGTs) and acyltransferases (ATs) further contribute to product diversification. PgUGT74AE2-mediated glycosylation enhances ginsenoside solubility [42], while LeSAT1 or LeAAT1-mediated acylation of shikonin with acetyl or isobutyryl groups enhances anticancer activity.

### 3.2. Medicinal Plant-Specific Regulatory Adaptations

Medicinal plants have evolved a remarkable diversity of species-specific adaptations to optimize the biosynthesis, storage, and sequestration of complex terpenoids. Key model species in which these adaptations have been extensively studied include *A*. *annua* (sesquiterpene lactone artemisinin), *Taxus* spp. (diterpenoid paclitaxel), *Salvia miltiorrhiza* (diterpenoid tanshinones), *Panax ginseng* (triterpenoid ginsenosides), and *C. roseus* (terpenoid indole alkaloids). The evolutionary strategies observed in these plants, often driven by gene duplication and functional diversification, provide a valuable framework for metabolic engineering by elucidating how nature has addressed the challenges of efficient, high-yield terpenoid production [43,44,45,46,47]. A central mechanism for expanding terpenoid diversity is lineage-specific gene duplication followed by functional diversification of biosynthetic enzymes [48]. This phenomenon is not limited to medicinal plants but represents a widely utilized evolutionary strategy. Maize (*Zea mays*) contains numerous terpene synthase (TPS) genes, with subtle active-site variations in functionally divergent enzymes such as TPS4 and TPS10 determining distinct sesquiterpene product specificities [49]. Within the genome of *Phaseolus lunatus*, two tandemly duplicated cytochrome P450 genes, *PlCYP82D47-like* and *PlCYP82D47*, are induced by herbivorous insects [50]. These enzymes catalyze the formation of volatile indirect defense signals, DMNT and TMTT, through the oxidative cleavage of terpenoid alcohol precursors [51]. The duplicated enzymes have undergone functional divergence, displaying distinct substrate preferences for (*E*)-nerolidol and (*E*,*E*)-geranyllinalool, respectively. Further molecular analyses reveal that key amino acid residues, such as L324 and L505 in the PlCYP82D47 enzyme, are essential for maintaining catalytic activity due to their critical roles in substrate recognition [50]. These residues thereby constitute the molecular basis of its substrate specificity.

The complex biosynthesis of many high-value terpenoids, such as paclitaxel, presents a significant compartmentalization challenge, requiring precise spatial organization across multiple organelles. This represents a critical consideration for synthetic biology approaches aiming to reconstitute these pathways in heterologous systems. A representative example is paclitaxel biosynthesis in *Taxus* species. The pathway initiates within plastids, where the plastid-targeted enzyme taxadiene synthase (TcTS) catalyzes the formation of the core hydrocarbon skeleton, taxa-4(5),11(12)-diene [52]. Following its synthesis, this hydrophobic intermediate must be translocated to the endoplasmic reticulum (ER). There, a series of membrane-bound cytochrome P450 enzymes (e.g., TcCYP725A4) carry out multiple oxidative modifications on the taxane ring [53,54]. Subsequent steps, including a critical C10-acetylation catalyzed by the soluble enzyme 10-deacetylbaccatin III 10-O-acetyltransferase (TcDBAT), occur in the cytosol [55]. Finally, to mitigate the self-toxicity of this potent compound, the mature paclitaxel molecule is exported from the cytoplasm and sequestered within the cell wall [56]. Critically, the transport mechanisms responsible for shuttling intermediates between plastids, the endoplasmic reticulum (ER), and the cytosol remain largely uncharacterized, although they may involve membrane contact sites [57,58]. Elucidating these mechanisms represents a major objective for both understanding native biosynthesis and facilitating efficient engineering in chassis platforms.

Subcellular compartmentalization and transport play crucial roles in plant secondary metabolism, where transporter proteins are essential. In *Salvia miltiorrhiza* (Danshen), the ATP-binding cassette (ABC) transporter SmABCG1, localized in the periderm cells of roots, has been shown to mediate the export of bioactive constituents, specifically tanshinones, into the extracellular space. A study employing CRISPR/Cas9 gene editing technology demonstrated that functional knockout of the *SmABCG1* gene led to a significant decrease in tanshinone content in both hairy roots and their culture medium. These findings highlight the indispensable role of SmABCG1 in the efficient secretion of tanshinones and the maintenance of normal metabolic flux [51]. Such regulatory mechanisms are also evident during the evolution of *Euphorbia* species. Research has demonstrated that peplusol synthase, which originated from the duplication of steroid synthase genes, enables the efficient heterologous production of linear triterpenoids in yeast, with yields reaching up to 30 mg/L [59].

In conclusion, the study of medicinal plants reveals a conserved set of adaptive strategies that synthetic biology can aim to emulate: (i) gene family expansion: Lineage-specific duplication of core biosynthetic genes generates genetic diversity that serves as a substrate for functional diversification, facilitating the evolution of novel chemical entities; (ii) subcellular compartmentalization: The coordinated organization of biosynthetic pathways across multiple organelles enables the utilization of distinct precursor pools and biochemical environments, while simultaneously mitigating cytotoxic effects. A major challenge in synthetic biology remains the identification and engineering of transporters responsible for shuttling metabolic intermediates between these compartments; (iii) spatial organization and sequestration: The development of specialized anatomical structures for the storage and secretion of terpenoids addresses the dual challenges of autotoxicity and efficient compound retrieval; (iv) regulatory specialization: The evolution of complex, often hierarchical, transcriptional and post-translational regulatory networks allows for precise spatial, temporal, and inducible control over extensive metabolic pathways. For metabolic engineers, these insights suggest that the successful heterologous production of complex terpenoids will likely require more than the mere reconstruction of enzymatic sequences. It may necessitate the recapitulation of spatial organization, the management of intracellular transport, and the implementation of dynamic regulatory systems to balance metabolic flux and prevent cellular toxicity.

### 3.3. Multilayer Regulatory Networks in Plant Systems

**Figure 2:**
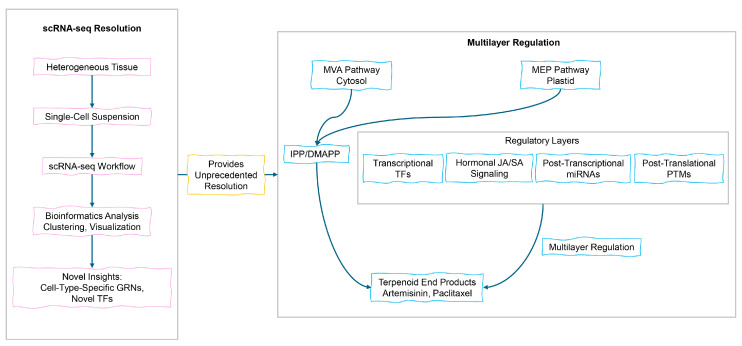
An integrated perspective on the multilayer regulatory network governing terpenoid biosynthesis and the dissection of this network through scRNA sequencing. Abbreviation: scRNA-seq, single-cell RNA sequencing; GRNs, gene regulatory networks; TFs, transcription factors; MVA Pathway, mevalonate pathway; MEP Pathway, methylerythritol phosphate pathway; IPP, isopentenyl pyrophosphate; DMAPP, dimethylallyl pyrophosphate; JA/SA, jasmonic acid/ salicylic acid; miRNAs, microRNAs; PTMs, post-translational modifications.

Transcription factors (TFs) play a central role in orchestrating terpenoid biosynthetic pathways and coordinating responses to diverse developmental and environmental signals. Key transcription factor families implicated in this regulatory process include AP2/ERF, bHLH, and MYB [60,61]. The jasmonate-responsive bHLH transcription factor AaMYC2 directly activates artemisinin biosynthesis by binding to the promoters of *CYP71AV1* and *DBR2*, with overexpression increasing artemisinin production by more than 2-fold [62]. Among MYB family members, AaMYB1 contributes to enhanced artemisinin production through the upregulation of *ADS* and *CYP71AV1* expression as well as increased glandular trichome density [63]. Hormonal crosstalk plays a critical role in fine-tuning specialized metabolism. Jasmonate (JA) induces artemisinin synthesis by promoting the degradation of Jasmonate ZIM-domain (JAZ) repressors, thereby derepressing transcriptional activators such as the TCP14-ORA complex. In contrast, salicylic acid (SA) antagonizes JA signaling at a downstream stage following JAZ degradation [64,65]. Non-coding RNAs contribute additional layers of regulatory control. *Cro-miR156a* mediates the cleavage of *CrSPL2/5* mRNAs, thereby repressing terpenoid indole alkaloid biosynthesis in *C*. *roseus* [66]. CRISPR-mediated editing of miR156 binding sites demonstrates potential for enhancing metabolic yields [67]. Similarly, artemisinin overproduction activates jasmonate signaling; the overexpression of key transcription factors enhances artemisinin synthesis by more than 2-fold [68]. Post-translational modifications (PTMs) dynamically regulate enzyme activity. Phosphorylation of HMGR at Ser-577 by SnRK1 results in enzyme inactivation, thereby modulating metabolic flux [69], while ubiquitination regulates the stability of transcription factors, the precise spatiotemporal control of terpenoid biosynthesis is ultimately achieved through a highly interconnected regulatory circuit. Within this network, transcriptional initiators, hormonal signals, non-coding RNAs, and post-translational modifiers collectively converge to fine-tune metabolic flux, thereby determining terpenoid yield and chemical diversity (Figure 2).

### 3.4. Engineering Regulatory Networks to Enhance Terpenoid Production

Recent advances in terpenoid biosynthesis have incorporated multiple engineering strategies to efficiently enhance production. Feedback inhibition represents a key engineering target. FPP accumulation inhibits HMGR activity; however, the expression of truncated yeast tHMGR effectively circumvents this limitation. In engineered yeast systems, the expression of tHMGR has been shown to increase terpenoid yields, elevating total sesquiterpenoids production by 36% and amorphadiene a precursor of artemisinin by fivefold [70,71]. Expression of soluble CrHMGR (from *C*. *roseus*) in *A. annua* elevates artemisinin levels by 22–38% [72]. CRISPR-Combo systems have the potential to enable dynamic pathway control in plant metabolic engineering. Studies have shown that simultaneous knockout of squalene synthase (SQS) and activation of terpene synthase (TPS) can redirect metabolic flux toward sesquiterpene production. Organelle engineering holds the theoretical potential to optimize plastid metabolic pathways by employing enzyme co-localization strategies analogous to synthetic protein scaffolds utilized in microbial systems [73]. Such approaches may reduce the diffusion of metabolic intermediates in the MEP pathway and potentially enhance IPP flux, although this hypothesis remains experimentally unvalidated in plastids [74]. These advances open new avenues for the derivatization of natural products, including Aconitum diterpenoids. In *S. cerevisiae*, the introduction of an isopentenol utilization pathway (IUP) enhances the supply of IPP/DMAPP [75]. Combined with metabolic engineering and process optimization, this strategy significantly enhances terpenoid production yields [76]. Collectively, these strategies exemplify the efficacy of modern biotechnological approaches in enhancing terpenoid biosynthesis (Figure 2).

### 3.5. Emerging Insights and Persistent Challenges

Single-cell omics has substantially enhanced spatial resolution in plant biology (Figure 2). This advancement is exemplified by scRNA-seq studies in Zea mays mesophyll cells, which identified 53 cell-type-specific transcription factors that regulate cell cycle dynamics during differentiation [77]. In *Cinnamomum camphora*, scRNA-seq studies have revealed dynamic transcriptional networks governing terpenoid biosynthesis, including 24 functionally annotated terpene synthase genes and 2863 differentially expressed genes between borneol- and camphor-type chemotypes [78]. While microbial and mammalian systems utilize identified IPP transporters, plant systems lack analogous, well-characterized transport mechanisms. Consequently, elucidating whether IPP crosses the chloroplast membrane through dedicated transporters or physicochemical gradients constitutes a fundamental priority in plant metabolic engineering [79]. In cytochrome P450 engineering, rational redesign of substrate pockets remains constrained by dynamic structural uncertainties [80]. Current progress increasingly arises from semi-rational strategies that integrate computational prescreening, thereby reducing the burden of mutant screening by over 95% compared to traditional approaches [81]. Additionally, the interplay between metabolic pathways and environmental stressors dynamically redistributes precursor allocation in planta, as quantified by isotopic flux studies. In *Arabidopsis thaliana*, phosphate stress reduces oxidative pentose phosphate pathway (oxPPP) flux by 38% while increasing anaplerotic flux through phosphoenolpyruvate carboxylase (PEPC) and malic enzyme, concurrently disrupting energy and nitrogen assimilation [82,83]. These responses exhibit tissue-specific divergence. Shoots prioritize photosynthetic phosphorus (Pi) remobilization, whereas roots accumulate organic acids for soil Pi chelation. Systematic multi-omics integration is therefore essential to resolve this complexity, as it has successfully linked dynamic metabolomes with transcriptional regulators to decode systemic adaptation mechanisms.

## 4. Metabolic Engineering Strategies Across Platforms

Metabolic engineering aims to reprogram cellular metabolism to enhance the production of valuable compounds, such as terpenoids. This objective necessitates a suite of strategies to overcome inherent regulatory bottlenecks and direct metabolic flux toward desired pathways. This section provides a comprehensive overview of these strategies, ranging from fundamental approaches such as overexpression of rate-limiting enzymes to advanced synthetic biology tools. The application and effectiveness of these strategies across native plants, heterologous plant hosts, and microbial chassis systems are discussed and compared.

### 4.1. Overexpression of Rate-Limiting Enzymes and Enhancement of Metabolic Pathways

For precursor supply in native plant systems, overexpression of key enzymes in the MVA/MEP pathway is crucial to enhancing artemisinin production in *A*. *annua*. Overexpression of key enzymes in the MVA/MEP pathway is crucial for enhancing artemisinin production in *A*. *annua*. Beyond the simple overexpression of native MVA pathway genes, advanced strategies include the expression of a truncated, feedback-insensitive HMGR (tHMG1) and the implementation of orthogonal pathways such as the isopentenol utilization pathway (IUP) to bypass endogenous regulatory mechanisms and enhance precursor supply [84]. Multi-gene co-expression strategies have demonstrated significant success, exemplified by the simultaneous overexpression of farnesyl diphosphate synthase (FPS), cytochrome P450 CYP71AV1, and its redox partner CPR, which together increased artemisinin yield by 3.6-fold in transgenic lines [85]. Similarly, co-expression of *amorpha-4,11-diene synthase* (*ADS*), *CYP71AV1*, *CPR*, and *aldehyde dehydrogenase 1* (*ALDH1*) resulted in a 3.4-fold increase by optimizing metabolic flux through the later biosynthetic steps [86]. Engineering of terpene synthases (TPS) has been accomplished through enzyme fusion approaches, as demonstrated by FPS-ADS fusion constructs that enhance substrate channeling and increase amorpha-4,11-diene production by 2- to 3-fold [68]. While precursor compartmentalization remains a challenge, co-overexpression of native cytosolic HMGR and plastidial DXR has been utilized to expand the overall precursor pool; however, plastid-targeting of HMGR has not been experimentally validated [87].

### 4.2. Precise Suppression of Competing Metabolic Pathways

Precise suppression of competing pathways represents a promising strategy for redirecting metabolic flux toward valuable plant metabolites, although specific applications require rigorous validation. For flux redirection, antisense suppression of SQS in tobacco has demonstrated efficacy by reducing sterol biosynthesis and increasing gibberellin GA3 production through the diversion of farnesyl pyrophosphate toward diterpenoid pathways [88]. Similarly, RNAi-mediated knockdown of SmHPPD in *Salvia miltiorrhiza* successfully enhanced rosmarinic acid and salvianolic acid B yields by reducing substrate competition [89]. Although CRISPR interference (CRISPRi) holds theoretical potential for such applications, its implementation for plant pathway suppression has not yet been experimentally validated. These cases highlight that effective flux redirection requires method-specific verification, with RNAi and antisense approaches currently representing the most well-documented strategies for competitive pathway suppression in plant systems [90].

### 4.3. Hierarchical Regulation of Transcription Factors (TFs)

Transcription factor hierarchical regulation optimizes metabolite biosynthesis through coordinated overexpression of positive regulators and suppression of repressors. In *A*. *annua*, overexpression of the AaMYB2 transcription factor co-activates key artemisinin pathway genes, thereby enhancing biosynthesis [91]. Similarly, overexpression of *AaTGA6* enhances the expression of the same gene set, thereby confirming the effectiveness of this co-activation strategy [92]. For repressor suppression, RNAi-mediated silencing of ZCT-family transcription factors in *C*. *roseus* has been investigated as a strategy to alleviate repression of terpenoid indole alkaloid biosynthesis, although functional redundancy complicates the outcomes [93]. To mitigate pleiotropic effects associated with constitutive expression, tissue-specific promoters can be employed. The *Agrobacterium rhizogenes*-derived RoIC promoter, which drives phloem-specific expression, enables spatial confinement of transcription factor activity, as demonstrated in transgenic potato systems [94].

### 4.4. Heterologous Pathway Reconstruction and Enzyme Engineering

**Figure 3:**
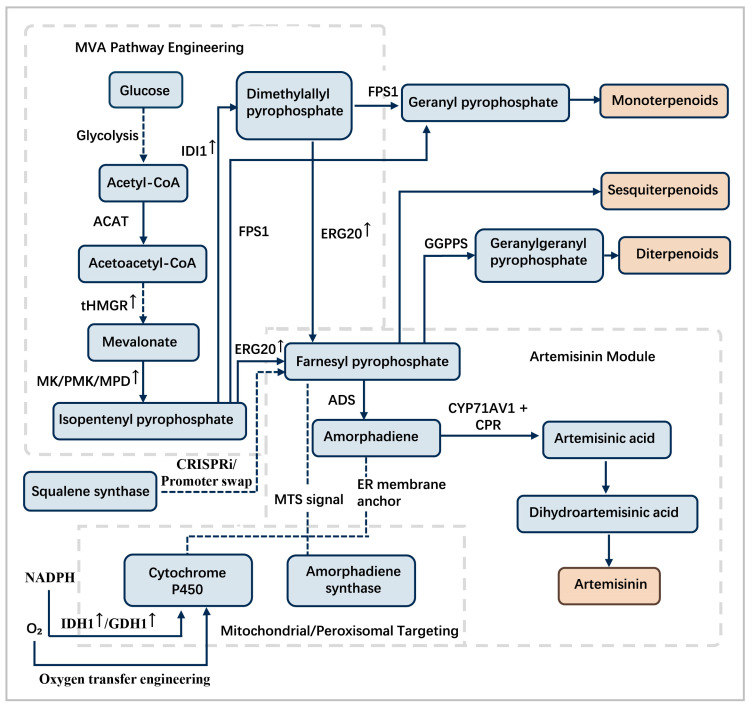
Strategies for optimized terpenoid production in *Saccharomyces cerevisiae*. This schematic illustrates the enhancement of terpenoid biosynthesis through metabolic engineering of the mevalonate (MVA) pathway. Abbreviations: tHMGR, truncated HMG-CoA reductase; IDI, isopentenyl diphosphate isomerase; FPS, farnesyl diphosphate synthase; ADS, amorphadiene synthase; IDH, isocitrate dehydrogenase; GDH, glutamate dehydrogenase; ERG20, farnesyl pyrophosphate synthase; GGPPS, geranylgeranyl pyrophosphate synthase; CPR, cytochrome reductase; MTS, mitochondrial targeting sequence. Legend: Blue blocks: Illustrate the MVA pathway and its metabolic intermediates. Orange blocks: Illustrate the end product of MVA pathway. Solid blue arrows: Indicate direct enzymatic conversions. Dashed blue arrows: Represent multi-step enzymatic processes. Dashed blue lines: Denote subcellular compartmentalization strategies. ↑: Indicates upregulation or engineering of native enzymes, including tHMGR [5], MK/PMK/MPD [16], IDH1/GDH1 [99], IDI1 and ERG20 [100].

The strategic rewiring of *S*. *cerevisiae* for terpenoid production involves a comprehensive synthetic biology approach. This includes host metabolic engineering, pathway reconstruction, enzyme engineering, and subcellular compartmentalization. Collectively, these strategies constitute an effective toolkit for overcoming metabolic bottlenecks and achieving high-level terpenoid production. Rewiring of the mevalonate (MVA) pathway in *S. cerevisiae* incorporates enzyme engineering, precursor redirection, and subcellular organization (Figure 3), systematically addressing metabolic limitations to enhance terpenoid biosynthetic flux. In *N*. *tabacum*, the taxadiene synthase (TS) gene from *Taxus brevifolia* was expressed in chloroplasts through the use of a chloroplast transit peptide. This strategy resulted in taxadiene yields of 87.8 µg/g dry weight by utilizing plastidial geranylgeranyl diphosphate (GGPP) pools for precursor supply [95]. For complex triterpenoid pathways, *S*. *cerevisiae* has proven to be an effective host. Co-expression of ginseng dammarenediol-II synthase (PgDDS) and cytochrome P450 CYP716A47 successfully enabled the synthesis of protopanaxadiol (PPD), which serves as the core ginsenoside backbone [96]. Further demonstrating cross-kingdom potential, tobacco plants co-expressing *PgDDS*, *CYP716A47*, and *CYP716A53v2* produced protopanaxatriol (PPT), thereby confirming the functional reconstitution of multi-step oxidation cascades [97]. These cases underscore the critical importance of precise enzyme selection, compartmentalization, and host compatibility for successful pathway transplantation. The successful reconstitution of metabolic pathways in yeast and plant systems exemplifies the broader trend of producing diterpenoid pharmaceuticals within optimized heterologous expression platforms [98].

Enzyme engineering plays a critical role in optimizing the kinetic properties, stability, and substrate specificity of terpenoid biosynthetic enzymes, particularly cytochrome P450s (CYPs) and glycosyltransferases (UGTs) [84]. Rational design utilizes high-resolution protein structures and computational simulations to identify key residues that govern substrate binding, regioselectivity, and catalytic efficiency. Studies have demonstrated that structure-guided mutations in the substrate access channels of taxadiene synthase (TS) not only alter product profiles but also increase tax-4(20),11(12)-diene yield through reprogramming of catalytic residues [101]. When structural data is limited, semi-rational strategies such as saturation mutagenesis at conserved sites or directed evolution are commonly employed [102]. This strategy effectively enhances the solubility and activity of membrane-anchored plant P450s in microbial hosts through N-terminal anchor truncation and GFP-fusion screening. Machine learning (ML) models trained on sequence-structure-activity relationships are now accelerating variant identification. For example, protein language models integrated with AlphaFold2 predictions enable highly accurate functional classification of terpene synthases and prediction of mutation impacts [102].

### 4.5. Directed Subcellular Metabolic Channeling

Subcellular targeting represents a sophisticated strategy for enhancing terpenoid biosynthesis by leveraging the distinct biochemical environments and precursor pools of cellular organelles [84]. This is achieved by genetically fusing enzymes to specific targeting signal peptides (SPs) that direct their translocation. Commonly used SPs include the N-terminal chloroplast transit peptide (cTP) for plastid import, the mitochondrial targeting sequence (MTS) for mitochondrial import, and the C-terminal endoplasmic reticulum (ER) retention signal for compartmentalization within the secretory pathway [84]. This approach not only positions enzymes in closer proximity to high-concentration precursor pools, but also reduces metabolic cross-talk and the potential cytotoxicity of intermediates by sequestering them from the cytosol [103]. Furthermore, co-targeting sequential enzymes to the same compartment facilitates metabolic channeling, whereby intermediates are directly transferred without diffusion, thus enhancing pathway efficiency and yield [103]. In *N. benthamiana*, plastid-targeted expression of monoterpene synthases using the RuBisCO small subunit transit peptide (TP) increased yields by more than 3-fold (compared to the original 1.2-fold) through access to concentrated plastidial IPP/DMAPP pools [84]. Mitochondrial targeting of sesquiterpene synthases in yeast through fusion to a COX4-derived mitochondrial targeting sequence (MTS) enhanced valencene production by 2.8-fold, confirming the organelle-specific advantages in redox cofactor supply [103]. Reconstruction of the entire cytosolic mevalonate (MVA) pathway in tobacco chloroplasts enhanced triterpenoid yields tenfold through compartmentalized channeling, demonstrating the efficacy of whole-pathway relocation [84]. Beyond targeting enzymes to classical organelles such as chloroplasts and mitochondria, innovative strategies in yeast involve engineering subcellular structures, including the endoplasmic reticulum (ER) and lipid droplets, to sequester and store hydrophobic terpenoid products, thereby reducing cytotoxicity and enhancing yield [84]. These strategies, together with other representative applications, illustrate how validated subcellular targeting can optimize metabolic flux through the strategic exploitation of spatial organization (Table 2). This is consistent with the emerging consensus that the strategic subcellular localization of enzymes and metabolic pathways represents a critical determinant of success in plant terpenoid engineering, frequently surpassing the impact of mere overexpression of pathway enzymes [23].

### 4.6. Cofactor Balancing and Dynamic Regulatory Mechanisms

Strategic cofactor management and dynamic pathway control are essential for optimizing terpenoid biosynthesis. In *A. annua*, NaCl-induced activation of glucose-6-phosphate dehydrogenase (G6PDH) enhanced NADPH regeneration under oxidative stress, which correlated with a 79.3% increase in artemisinin production [107]. For dynamic control, methyl jasmonate (MeJA)-responsive promoters drive coordinated tanshinone biosynthesis in *Salvia miltiorrhiza*, with SmMEC gene expression increasing more than 5-fold following elicitation [108]. This endogenous regulatory system enables the synchronized upregulation of pathway genes (*SmHMGR*, *SmDXR*, *SmGGPPS*), while RNAi-mediated knockdown of JAZ repressors enhances tanshinone yields by 2- to 3-fold through the derepression of MYC2 transcription factors [109]. Complementary approaches, such as engineered NADH-dependent HMGR variants in microbial systems, further alleviate NADPH bottlenecks without compromising redox balance [110].

### 4.7. Advances in Synthetic Biology Tools

The mechanistic foundation of CRISPR-based tools in plants relies on the formation of a Cas protein-guide RNA (gRNA) complex that induces site-specific double-strand breaks (DSBs) [111]. These breaks are subsequently repaired by the plant’s endogenous repair machinery, primarily via error-prone non-homologous end joining, resulting in gene knockouts, or, less commonly in plants, through homology-directed repair to achieve precise genetic edits [112,113]. Advancements now extend beyond the canonical CRISPR-Cas9 nuclease to encompass base editors (BEs), which catalyze C:G-to-T:A or A:T-to-G:C conversions without inducing double-strand breaks (DSBs) [114], and prime editors (PEs), which are capable of introducing all 12 possible base-to-base conversions, as well as small insertions and deletions, through the use of a reverse transcriptase-encoded template [115]. Delivery methods, such as Agrobacterium-mediated T-DNA integration or the direct delivery of pre-assembled ribonucleoprotein (RNP) complexes, are key determinants of editing efficiency and off-target effects [116,117]. Although applications in plants predominantly focus on gene knockouts, emerging strategies employ CRISPR activation (CRISPRa) systems that utilize deactivated Cas9 (dCas9) fused to transcriptional activators such as VP64 or EDLL to upregulate key biosynthetic genes [118].

Recent advances in synthetic biology have substantially advanced metabolic engineering in medicinal plants, particularly through CRISPR-based multiplex genome editing. Validated CRISPR-Cas9 systems enable the efficient simultaneous knockout of multiple gene targets in hairy root cultures, which serve as a critical functional genomics platform for recalcitrant species. Studies have demonstrated that, in *Eucalyptus grandis*, dual sgRNA constructs achieved a 75% editing efficiency for concurrent mutation of *EgrCCR1* and *EgrIAA9A* [119]. Furthermore, studies have demonstrated that codon-optimized Cas9 systems in soybean enable precise multiplex editing of homeologous genes. In the context of metabolic pathway regulation, engineered promoters now allow for tissue-specific and inducible terpenoid biosynthesis, thereby mitigating growth defects associated with constitutive overexpression [67]. Delivery systems have also advanced: optimized *Agrobacterium* strains combined with surfactants have achieved a transformation efficiency of 70.9%, and hairy root cultures consistently overcome regeneration bottlenecks in slow-growing species such as *Panax ginseng*, resulting in 2- to 3-fold higher metabolite titers. Nevertheless, persistent challenges remain, including genotype-dependent regeneration and the necessity to validate emerging tools such as CRISPR-Combo systems and nanoparticle co-delivery approaches in medicinal plant species.

### 4.8. Critical Analysis of Metabolic Engineering Strategies: Mechanistic Insights into Success and Failure

#### 4.8.1. The Paradigm Shift from ‘Rate-Limiting Steps’ to Distributed Metabolic Control

The field of metabolic engineering has undergone substantial evolution, shifting from an initial emphasis on single-gene overexpression to more advanced, systems-level approaches. Early efforts to enhance metabolic pathways through the overexpression of a single “rate-limiting” enzyme frequently produced inconsistent outcomes. This phenomenon is well explained by Metabolic Control Analysis (MCA), which demonstrates that control over metabolic flux is generally distributed among multiple enzymes rather than being localized to a single step [120,121,122]. Consequently, simply enhancing the activity of a single enzyme may shift the metabolic bottleneck to downstream steps, resulting in only modest improvements in product yield and potentially leading to the accumulation of toxic intermediates. This is a consequence commonly observed when gene expression levels are not appropriately balanced [84,123].

#### 4.8.2. Precursor Availability as a Critical Metabolic Bottleneck: The Case of ADS in Artemisia Annua

Following the recognition that single-enzyme overexpression is often insufficient to enhance metabolic flux, researchers began to acknowledge the essential role of substrate and precursor availability in determining pathway efficiency. A prominent example of this limitation is observed in the artemisinin biosynthesis pathway in *A. annua*. Although overexpression of amorpha-4,11-diene synthase (ADS), the enzyme catalyzing the first committed step in the pathway, has been widely implemented, its effectiveness is frequently constrained by the limited availability of precursors, can enhance metabolic flux towards artemisinin precursors, the effectiveness of this strategy depends on the availability of essential substrates, such as farnesyl diphosphate (FPP) [111,124]. Experimental evidence strongly supports this dependency. Research has demonstrated that silencing ADS results in the accumulation of its substrate, farnesyl diphosphate (FPP), indicating that the supply of FPP can surpass the capacity of a non-engineered downstream pathway [125]. Conversely, successful strategies to substantially increase artemisinin content have frequently involved the concurrent overexpression of ADS together with an upstream gene responsible for FPP synthesis, such as HMG-CoA reductase (HMGR) or farnesyl diphosphate synthase (FPS) [26,126]. This highlights the importance of addressing precursor availability in conjunction with enzyme activity to achieve substantial increases in product accumulation.

#### 4.8.3. Pathway Balancing Through Attenuation of Competing Metabolic Fluxes

A more sophisticated strategy involves the active redirection of metabolic resources by suppressing competing pathways. This approach represents a significant advancement over the conventional method of attempting to enhance flux through the target pathway in isolation. To overcome the limitations associated with single-gene overexpression, a more effective approach focuses on pathway balancing, which involves the attenuation of competing metabolic fluxes. In the context of terpenoid biosynthesis, branch-point metabolites such as farnesyl diphosphate (FPP) and geranylgeranyl diphosphate (GGPP) function as substrates for multiple essential pathways, including those responsible for the synthesis of sterols, carotenoids, and hormones [125,127]. Engineering efforts that overlook these competing pathways are unlikely to succeed, as the native cellular machinery will continue to redirect precursors away from the desired pathway. A significant milestone in this field was the downregulation of the sterol biosynthesis pathway in *A. annua* through RNA interference (RNAi) targeting SQS [128,129]. SQS catalyzes the first committed step in the sterol biosynthesis pathway, thereby directly competing with ADS for the same pool of FPP precursor [130,131]. This intervention effectively redirected FPP towards the artemisinin biosynthesis pathway, resulting in a significant increase in artemisinin content.

#### 4.8.4. Integrated Multi-Gene Engineering: The “Push, Pull, and Block” Strategy

The culmination of these insights has led to integrated, multi-faceted engineering strategies that simultaneously target multiple nodes within a metabolic network. The “push, pull, and channel/block” concept provides a valuable framework for describing such complex interventions. “Push” refers to enhancing precursor supply, “pull” denotes the promotion of target product formation, and “block” signifies the down-regulation of competing pathways [132]. The most significant advancements in terpenoid production have arisen from multi-gene engineering approaches that concurrently enhance precursor supply and downstream conversion capacity. This coordinated strategy prevents the accumulation of toxic intermediates and establishes a metabolic sink that directs metabolic flux towards the final product. In *A. annua*, the co-expression of multiple pathway genes, including amorpha-4,11-diene synthase (ADS, “pull”), a cytochrome P450 monooxygenase (CYP71AV1, representing an additional “pull” component), and its redox partner (CPR), is often combined with upstream engineering (e.g., HMGR overexpression for “push”) [26], has led to considerable improvements in artemisinin yield. Furthermore, the application of tissue-specific promoters enables the localization of metabolic burden [133]. This strategy enhances the efficient allocation of precursors and minimizes growth-associated penalties.

#### 4.8.5. Analysis of Persistent Challenges in Terpenoid Metabolic Engineering

Despite advances in multi-gene engineering, progress remains limited due to persistent challenges inherent to cellular complexity. Even sophisticated interventions may fail for the following key reasons: (i) residual Regulatory Complexity and Feedback Inhibition: Terpenoid biosynthetic pathways, such as the MEP pathway, are subject to stringent, multi-layered regulation at both the genetic and allosteric levels [134]. Overcoming one bottleneck may result in the accumulation of an intermediate metabolite, which can subsequently induce feedback inhibition of an upstream pathway enzyme, thereby creating a new, unforeseen bottleneck [84,135]. These native regulatory circuits can attenuate or negate the effects of even robust multi-gene expression. (ii) metabolic Burden and Intermediate Toxicity: The high-level expression of multiple heterologous genes and the forced redirection of major carbon flux can impose a significant metabolic burden on the host organism, adversely affecting growth and cellular viability [136]. Furthermore, an imbalanced pathway, in which a “push” strategy is not complemented by an adequate “pull,” can result in the accumulation of pathway intermediates that are cytotoxic, thereby impairing cellular function and ultimately constraining product yield [17,137]. (iii) inherent Catalytic Limitations and Enzyme Promiscuity: Many key enzymes involved in terpenoid biosynthesis, particularly terpene synthases, demonstrate low catalytic efficiency or exhibit high promiscuity, leading to the concurrent production of a range of side-products along with the desired molecule [137]. Protein engineering can mitigate this challenge to a certain extent; however, the intrinsic properties of these enzymes continue to represent a fundamental barrier to achieving high titers and purity. (iv) lack of Precise Genetic Tools and Fine-Tuning: Although powerful, existing genetic tools frequently fall short in their capacity to precisely and stably modulate the expression levels of multiple genes simultaneously [138]. Achieving the optimal stoichiometric balance among multiple enzymes within a metabolic pathway represents a significant challenge, often necessitating extensive combinatorial screening of gene expression levels. This task is particularly demanding in complex organisms such as plants. (v) insufficient Knowledge and Unpredictable System Behavior: A primary obstacle remains our incomplete understanding of the entire metabolic network, encompassing the kinetic parameters of all relevant enzymes and the complex cross-talk between pathways [135,138]. Engineering a target pathway can exert profound and unforeseen effects on off-target pathways via metabolite signaling, resulting in system-wide perturbations that are challenging to anticipate and manage.

## 5. Genomics and Multi-Omics: Elucidating the Blueprint and Potential Targets

### 5.1. Foundation: Genome Sequencing and Gene Identification

Advancements in genome sequencing have become foundational for the identification of genes involved in terpenoid biosynthesis, with high-quality chromosome-level genomes playing a critical role. Recent studies have employed long-read sequencing technologies, such as PacBio HiFi and Oxford Nanopore, in conjunction with Hi-C chromatin mapping, to assemble the complex and repetitive genomes of medicinal plants with high contiguity (N50 > 10 Mb) [139,140,141,142]. These methodologies enable the identification of key gene families, such as Terpene Synthase (TPS), Cytochrome P450 (CYP), and UDP-Glycosyltransferase (UGT), primarily through homology-based screening and conserved domain analysis. In *A. annua*, chromatin conformation analysis has uncovered spatial interactions that regulate the artemisinin biosynthetic cluster [143]; synteny analysis remains the primary method for identifying evolutionarily conserved clusters across species [144]. Comparative genomics further offers insights into evolutionary dynamics by elucidating expansion and contraction events within gene families, thereby enhancing our understanding of terpenoid diversification [145,146,147].

### 5.2. Decoding Dynamic Processes: Transcriptomic and Metabolomic Profiling

Bulk RNA sequencing plays a crucial role in delineating tissue-specific expression patterns of core terpenoid biosynthetic genes. Notably, this includes Terpene Synthases (TPS) and Cytochrome P450s (CYPs) in specialized structures such as glandular trichomes and roots. This approach also effectively captures inducible expression dynamics, particularly in response to jasmonate signaling, as demonstrated by the rapid upregulation of sesquiterpene synthases in tomato trichomes and defense-related Cytochrome P450s in *N. attenuata*. [148,149]. Weighted Gene Co-expression Network Analysis (WGCNA) identifies transcriptional modules that are strongly correlated with terpenoid accumulation and proposes candidate regulatory transcription factors for experimental validation of cis-element interactions [150,151]. The emergence of single-cell and spatial transcriptomics offers transformative potential for associating gene expression profiles with specific cell types and anatomical regions. A study utilizing scRNA-seq identified enrichment of terpenoid biosynthesis genes in epidermal cell subpopulations [152]. Spatial transcriptomics enables mapping at cellular resolution in model plants, as exemplified by MERFISH-based visualization of auxin transporters in the leaf vasculature of *A. thaliana* [153].

High-resolution mass spectrometry techniques, particularly liquid chromatography-tandem mass spectrometry (LC-MS/MS) and gas chromatography-mass spectrometry (GC-MS), are essential for untargeted profiling of complex terpenoids across diverse plant taxa. These methods facilitate robust correlations between metabolite abundance and gene expression when integrated with Weighted Gene Co-expression Network Analysis (WGCNA), as illustrated in studies of *Gynostemma pentaphyllum* and *Ferula assafoetida* [151,154]. Although ion mobility spectrometry (IMS) offers additional separation capabilities for resolving isomers, its application is primarily confined to metabolite identification and has seen limited use in gene-metabolite correlation studies [155]. The established HRMS-WGCNA framework supports metabolic engineering evaluations by quantifying titer improvements. It demonstrates increased sesquiterpene lactone levels in engineered *A. annua* and quantifies reductions in byproducts within engineered systems [156,157]. In *Centella asiatica*, recent advances in machine learning have improved the quantification of known saponins such as asiaticoside, while the discovery of novel saponins has continued to rely on classical NMR and HRMS approaches [158]. Crucially, the functional roles of UDP-glycosyltransferases in saponin biosynthesis are increasingly being elucidated through biochemical characterization [158].

### 5.3. Beyond Abundance: Proteomic and Epigenomic Regulatory Mechanisms

Emerging quantitative proteomics techniques, such as Tandem Mass Tag (TMT) and Sequential Window Acquisition of All Theoretical Mass Spectra (SWATH-MS), show considerable potential for comprehensive profiling of enzyme abundance in terpenoid biosynthesis, although their standardized application specifically to terpenoid pathway enzymes remains to be further developed [159,160]. Complementing abundance measurements, phosphoproteomics provides critical insights into kinase-mediated regulatory mechanisms. A well-established and experimentally validated example is the inhibitory phosphorylation of 3-hydroxy-3-methylglutaryl-coenzyme A reductase (HMGR), the rate-limiting enzyme in the mevalonate (MVA) pathway, at serine 577 in *A. thaliana*, which directly decreases its enzymatic activity [161,162]. Targeted proteomic approaches, such as Selected Reaction Monitoring (SRM), have been effectively utilized to quantify dynamic changes in the abundance of terpenoid biosynthetic enzymes, including terpene synthases (TPS) and DXS isoforms, under elicitation conditions [163]. Phosphorylation-mediated regulation of cytochrome P450 (CYP450) enzymes constitutes a well-established regulatory mechanism in biological systems [164]. Phosphorylation of human CYP450c17 by p38α kinase enhances the enzymatic activity of its 17,20-lyase domain during androgen biosynthesis [165]. These findings underscore the importance of rigorously investigating post-translational modifications, particularly phosphorylation, in the regulation of terpenoid biosynthesis and highlight both validated and potential targets for metabolic engineering.

Chromatin-level regulation, encompassing DNA methylation, histone modifications, and three-dimensional conformation, is theorized to influence terpenoid gene expression, although direct mechanistic evidence remains limited. Empirical studies have confirmed that DNA methylation actively modulates terpenoid biosynthetic pathways. In *Rehmannia glutinosa*, treatment with the demethylating agent 5-azacytidine resulted in the upregulation of iridoid glycoside biosynthesis genes and a 2.3-fold increase in monoterpene accumulation [166]. In *Eleutherococcus senticosus*, hypermethylation was shown to suppress saponin biosynthetic genes, resulting in a 60% reduction in triterpenoid content [166]. Techniques such as ChIP-seq and ATAC-seq have the potential to advance our understanding of histone modifications in terpenoid-producing tissues, particularly in light of established histone marks in other specialized metabolic pathways. These findings reveal substantial gaps in current knowledge regarding the epigenomic regulation of terpenoid biosynthesis and underscore the necessity for targeted studies aimed at harnessing the potential of epigenetic engineering.

### 5.4. Systems Integration: Constructing Predictive Models for Engineering Applications

Integrating genomics, transcriptomics, metabolomics, and epigenomics through systems biology approaches is crucial for advancing predictive engineering in plant biotechnology. By constructing gene regulatory networks (GRNs) and genome-scale metabolic models (GEMs), researchers can identify key regulatory elements, such as MYB and bHLH transcription factors, as well as rate-limiting enzymes that are essential for optimizing metabolic pathways. Machine learning techniques enable comprehensive analysis of multi-omics datasets, facilitating the prediction of gene functions and the identification of optimal engineering targets. For instance, Random Forest regression has been successfully employed to integrate transcriptomic and metabolomic data in potato, enabling the prediction of phenotypic traits associated with tuber development [167]. Similarly, MYB and bHLH transcription factors, which are known to regulate phenylpropanoid and flavonoid biosynthesis through MBW complexes, have been utilized to enhance anthocyanin production in model plant systems [168]. These predictive frameworks serve as the foundation for synthetic biology design, enabling the construction of minimal gene circuits for heterologous production in host systems such as yeast or *N. benthamiana*, as exemplified by the reconstruction of benzylisoquinoline alkaloid pathways [169]. While the transformative potential is evident, current advancements primarily focus on the iterative refinement of models utilizing 2 to 4 omics layers, with pioneering studies on elicitor-induced paclitaxel enhancement laying the groundwork for future multi-target engineering strategies [170].

## 6. Biotechnological Applications: From Laboratory Research to Prospective Industrialization

### 6.1. High-Yielding Cultivation of Medicinal Plants and Cell Lines

Metabolic engineering has substantially advanced high-yielding terpenoid production in medicinal plants, with multiple experimentally validated breakthroughs. In *A. annua*, the stacked overexpression of *ADS*, *CYP71AV1*, *ALDH1*, and *POR* genes resulted in a 3.4-fold increase in artemisinin content, surpassing earlier single-gene strategies [26,85]. While stacking key biosynthetic genes enhances titers, dynamic flux control is essential to mitigate metabolic imbalances in scaled systems [171,172]. Machine learning-driven predictive modeling can identify rate-limiting steps that are not addressable through conventional enzyme overexpression. The integration of transcriptomic and metabolomic data through Random Forest regression enables in silico prediction of terpenoid accumulation patterns under varying nutrient conditions [173,174]. Coupled with optogenetic switches (e.g., blue-light-activated systems and OptoAMP gene circuits in yeast), this approach enables real-time redirection of carbon flux toward target pathways, thereby improving both yield and stability during extended cultivation periods [74,171,175]. For paclitaxel, optimized plant cell suspension cultures continue to represent the most productive system, achieving industrially validated titers of 900 mg/L, whereas microbial platforms have so far been limited to precursor synthesis only [176]. Equally significant is the co-expression of *SmCPS1* and *SmKSL1* in *Salvia miltiorrhiza* hairy roots, which robustly enhances tanshinone IIA accumulation, although quantitative genetic stability metrics across subcultures remain to be fully documented [177]. Recent advances in CRISPR technology include the modulation of trichome density in *A. annua* through SGS3 gene editing, a validated strategy for indirectly increasing artemisinin levels [131].

### 6.2. Production of Rare and Structurally Complex Terpenoids

Plant chassis systems offer distinct advantages for the synthesis of highly oxidized terpenoids through the utilization of native cytochrome P450 (CYP) enzymes and subcellular compartmentalization. A well-validated example is the production of artemisinic acid in *N. benthamiana* via heterologous expression of *ADS* and *CYP71AV1*, demonstrating the system’s capability to perform complex oxidation reactions [21]. For triterpenoid saponins, *Panax ginseng* engineered through CRISPR-mediated suppression of *CYP716A53v2* combined with phenylalanine ammonia-lyase (PAL) overexpression produced ginsenoside Rg3 at 7.0 mg/g dry weight. This represents a 21-fold increase compared to the wild type, marking the highest plant-derived yield documented to date [178].

However, cytotoxicity caused by reactive intermediates remains a significant bottleneck. Synthetic biology approaches provide potential solutions through the following strategy: (i) subcellular channeling utilizing a synthetic protein scaffold that regulates metabolic flux via modular control, optimizing the enzyme ratios in the mevalonate pathway in *Escherichia. coli* and preventing the toxic accumulation of HMG-CoA, thereby enhancing the yield by 77-fold [179,180]; and (ii) heterologous expression of detoxifying enzymes such as epoxide hydrolases, which has been shown to reduce cytotoxicity in engineered *E. coli* [181,182]. These strategies effectively minimize cellular damage while simultaneously enhancing pathway efficiency. Microbial systems remain superior for certain glycosylation steps, as demonstrated by yeast platforms expressing *PgUGT74AE2* and *PgUGT94Q2* that achieve yields exceeding 250 mg/L of Rg3 [183]. These cases underscore the potential of plant chassis systems while highlighting context-specific limitations in the biosynthesis of rare terpenoids.

### 6.3. Biosynthesis of Novel Terpenoid Derivatives

Combinatorial biosynthesis has significantly expanded the repertoire of “non-natural natural products” by enabling systematic diversification of terpenoid scaffolds. Engineered CYP76-family enzyme libraries (CYP76AH/CYP76AK) expressed in yeast have successfully oxidized abietadiene precursors to yield 14 abietane diterpenes [184]. Among these, eight were previously unreported, demonstrating unprecedented scaffold diversity. Notably, novel compounds such as Liquidambarines A-C exhibit potent anti-inflammatory activity through the suppression of NF-κB-mediated iNOS and COX-2 expression in macrophages [21]. Concurrently, the modular assembly of terpene synthases and P450 enzymes in yeast enables artificial triterpene biosynthesis, as exemplified by functional partnerships such as β-amyrin synthase paired with CYP716Y1 to generate C-16α-hydroxylated derivatives [185]. These approaches highlight the capability to engineer structurally complex terpenoids with precisely tailored bioactivities.

### 6.4. Enhanced Plant Stress Tolerance

Terpenoid metabolic engineering provides promising strategies for enhancing plant stress resilience. Overexpression of the *TPS10* terpene synthase in *A. thaliana* significantly deterred aphids through the emission of linalool and other volatile compounds, as demonstrated in controlled dual-choice assays with robust statistical validation [186]. Similarly, in *Salvia miltiorrhiza*, overexpression of specific *SmJAZ* isoforms (*SmJAZ1/2/5/6*) activated jasmonate signaling and increased tanshinone accumulation through the upregulation of diterpenoid biosynthetic genes (*SmGGPPS*, *SmKSL*) [187]. These examples underscore both the potential and the limitations of terpenoid engineering, highlighting the necessity for isoform-specific characterization and rigorous statistical reporting.

### 6.5. Plant Cell and Tissue Culture: Challenges in Scale-Up and Industrial Application

Suspension cell culture combined with bioreactor technology provides a feasible platform for the industrial-scale production of high-value compounds such as paclitaxel and ginsenosides. For paclitaxel, validated bioreactor yields have reached 25.63 mg/L in 20 L-scale systems [188]. However, scale-up encounters significant challenges, including genetic instability in cell lines, which compromises long-term yield consistency. Notably, commercial-scale production has been successfully achieved by companies such as Phyton Biotech and Samyang Genex through the application of plant cell culture technology [189]. For ginsenosides Rg3, engineered yeast systems exhibit significant potential, achieving yields of 254.07 mg/L in shake flasks; however, documented bioreactor production at scales exceeding 100 L has not yet been reported [190]. Key scale-up barriers encompass shear stress sensitivity in plant cells and the requirement for dynamic nutrient regulation [191]. Addressing the economic challenges of scale-up necessitates the integration of multi-omics approaches with AI-driven optimization. Media design guided by multi-omics data, correlating nutrient consumption with paclitaxel yields through LC-MS/MS and RNA-seq analysis, has significantly reduced byproduct formation in *Taxus* bioreactors [17,192]. Concurrently, AI algorithms integrated with real-time sensors enable dynamic adjustment of dissolved O_2_ and pH, achieving a productivity of up to 0.88 mg/g DCW/day in perfusion bioreactor systems [193]. Such closed-loop control minimizes operational expenditures while substantially lowering production costs [194].

### 6.6. Plant-Based Systems as Sustainable Cell Factories

Compared to microbial fermentation, plant-based systems offer inherent advantages for the biosynthesis of complex terpenoids due to the presence of fully functional endogenous enzyme systems. This is exemplified by the intricate P450 oxidation networks that enable terpenoid indole alkaloid biosynthesis in *C. roseus* [195]. Subcellular compartmentalization further prevents the accumulation of toxic intermediates, as illustrated by the storage of artemisinin in glandular trichome vesicles [111]. Additionally, native plant UGTs provide regioselective glycosylation that is essential for bioactivity [196]. However, heterologous expression of identical UGT isoforms in yeast results in reduced catalytic efficiency, primarily due to low expression levels, insufficient availability of UDP-sugar donors, and suboptimal kinetic properties [197]. As metabolic engineering progresses toward industrial-scale applications, CRISPR-based genome editing demonstrates potential for targeted pathway optimization; however, the long-term genetic stability in suspension cultures remains to be validated [198]. Similarly, although AI-sensor fusion is emerging as a tool for bioreactor control, its integration into industrial workflows remains limited due to challenges in real-time data processing and system compatibility [199], Currently, no industrial-scale platforms are available for the production of plant-derived terpenoids [200]. Beyond theoretical sustainability, engineered photoautotrophic chassis organisms demonstrate practical advantages. In *Chlamydomonas reinhardtii*, chloroplast-localized terpenoid pathways access endogenous plastidial IPP pools, resulting in β-carotene accumulation of up to 30.65 mg/g dry weight under photoautotrophic growth conditions [134]. Engineering overexpression of the bicarbonate transporter HLA3 enhanced inorganic carbon uptake, resulting in substantial increases in carbon fixation flux and precursor availability [201,202,203,204]. This chassis eliminates the need for organic feedstocks and substantially reduces terpenoid-induced cytotoxicity through inherent subcellular compartmentalization.

In conclusion, the most promising production system is not a universal solution; rather, it is critically dependent on the structural complexity of the target terpenoid and the primary economic drivers (e.g., cost, volume, and speed). Looking ahead, the most promising paradigm lies not in the dominance of a single platform, but in the development of integrated ‘smart’ systems. Such systems would harness the strengths of each platform for example, employing microbial systems for scalable precursor synthesis, followed by in vitro or in planta biotransformation for complex structural modifications guided by predictive models derived from systems and synthetic biology to optimize the entire value chain.

## 7. Current Challenges and Limitations

### 7.1. Complex Metabolic Pathways and Incompletely Characterized Regulatory Mechanisms

Terpenoid biosynthetic pathways are often highly branched, involving multi-step enzymatic cascades with promiscuous intermediate metabolites and extensive crosstalk with primary metabolic networks [205]. Regulatory networks that control metabolic flux remain largely uncharacterized in non-model medicinal species, as illustrated by the poorly understood transcriptional regulation of terpenoid indole alkaloids in *C. roseus* [205]. Advanced techniques are uncovering spatial regulatory complexity: In *A. annua*, transcriptomic analysis of laser-microdissected glandular trichome cell layers has revealed cell-type-specific expression divergence among artemisinin pathway genes [206]. Similarly, CRISPR/Cas9-mediated knockout of *CYP716A53v2* in *Panax ginseng* cell cultures successfully abolished specific ginsenoside production, thereby demonstrating precise dissection of the metabolic pathway [207].

### 7.2. Limitations in Genetic Transformation and Regeneration Efficiency

The genetic modification of many high-value medicinal plants is hindered by low transformation efficiency and technically demanding regeneration protocols. Species such as *Taxus* and *Catharanthus* often require prolonged de novo regeneration processes that exceed one year, in stark contrast to model systems like *N. benthamiana* [208]. Recent advances provide targeted solutions: in *Camptotheca acuminata*, optimized *Agrobacterium*-mediated transformation achieves 6% efficiency through refined co-cultivation parameters [209]; hairy root CRISPR systems in *Salvia miltiorrhiza* enable efficient editing, with reported efficiencies reaching 71.07% [210]; nodal section transformation in *Stevia rebaudiana* yields 40.48% efficiency [211]. While morphogenic transcription factors demonstrate transformative potential in recalcitrant crops such as maize [212], these collective advances provide validated strategies for the genetic manipulation of medicinal plant species.

### 7.3. Metabolic Imbalance, Growth-Associated Penalties, and Cellular Toxicity

Engineered hyperaccumulation of terpenoids frequently incurs metabolic trade-offs, primarily due to competition for central metabolites such as carbon skeletons, ATP, and NADPH, which can directly impair plant growth. This phenomenon has been empirically demonstrated in transgenic *Arabidopsis* lines expressing sesquiterpene synthases, where precursor depletion is correlated with significant growth retardation [213]. Although chloroplast-targeted sesquiterpene production has been observed in *Nicotiana* species [214], there is no direct evidence confirming that sesquiterpene overproduction disrupts chloroplast integrity via ROS bursts in engineered *N. benthamiana*. In contrast, chloroplast-derived ROS generation is a well-documented stress response under pathological or metabolic perturbations [215]. Reactive intermediates such as epoxides, which may be theoretically generated during taxadiene hydroxylation by cytochrome P450s, could pose toxicity risks if they accumulate. To mitigate potential instability, spatial sequestration or co-expression of detoxifying enzymes has been proposed as a potential safeguard [216]; however, its efficacy in planta remains to be validated.

### 7.4. Compartmentalization and Transport Limitations

Terpenoid biosynthesis involves multiple organelles, which inherently complicates the trafficking of metabolic intermediates across cellular membranes. Engineering efficiency is often constrained by the lack of characterized transporters; however, recent discoveries are beginning to overcome these limitations. In *C. roseus*, CrNPF2.9 has been identified as the transporter responsible for exporting the monoterpene indole alkaloid (MIA) intermediate strictosidine from vacuoles to the cytosol [217]. ABC transporters in *C. roseus* have been shown to facilitate the export of late-stage MIA metabolites across the plasma membrane [218]. Additionally, breakthroughs in chloroplast compartmentalization in *N. benthamiana* demonstrate transformative potential [219]. Targeting taxadiene synthase to chloroplasts resulted in the accumulation of taxadiene at a concentration of 56.6 μg/g fresh weight, representing an increase of several thousand-fold compared to the baseline level. These advancements highlight how the elucidation of transport mechanisms and optimization of subcellular targeting can effectively overcome critical bottlenecks in terpenoid metabolic engineering.

### 7.5. Suboptimal Enzymatic Characteristics

Protein engineering plays a crucial role in the optimization of key enzymes. For example, structure-guided mutations in farnesyl pyrophosphate synthase (Erg20p) have been successfully utilized to modify product specificity and increase the accumulation of geranyl diphosphate (GPP), a critical precursor for monoterpenes [84]. Key terpenoid-modifying enzymes, such as cytochrome P450s (CYPs) and UDP-glycosyltransferases (UGTs), often exhibit suboptimal characteristics, including low catalytic efficiency, structural instability, and substrate promiscuity, which collectively constrain the overall efficiency of terpenoid biosynthetic pathways. Recent advances in enzyme engineering, particularly directed evolution and machine learning (ML), have effectively addressed these limitations with demonstrable success [219]. ML-guided engineering of norbelladine 4′-O-methyltransferase through a structure-based residual neural network has yielded variants exhibiting a 60% improvement in product titer, a 2-fold enhancement in catalytic efficiency, and a 3-fold reduction in off-product formation [220]. In *Salvia miltiorrhiza*, the co-expression of upstream pathway genes *SmGGPPS* and *SmHMGR* resulted in a statistically validated 5.7-fold increase in tanshinone yield [221]. These engineering advancements highlight the potential of computational and combinatorial strategies to alleviate enzymatic bottlenecks in terpenoid biosynthesis.

### 7.6. Absence of Universal Chassis Plant Systems

Although *N. benthamiana* is widely used for transient expression studies, its applicability for sustained terpenoid production is limited by inherent metabolic constraints and carbon partitioning competition, thereby necessitating the development of specialized chassis plants. Recent advances in genetic engineering provide promising alternatives; for example, CRISPR/Cas9-mediated knockout of SQS in *A. annua* achieved an 84.6% mutagenesis efficiency, effectively redirecting metabolic flux toward artemisinin precursors and increasing yields up to threefold [26]. Furthermore, hairy root cultures of *Ophiorrhiza pumila* enable stable camptothecin production at concentrations ranging from 0.1% to 0.3% of dry weight; however, bioreactor scalability has yet to be validated at pilot scale [222]. These strategies represent significant progress toward the development of customized plant chassis systems; however, further optimization is required to achieve sustainable and scalable terpenoid biosynthesis.

### 7.7. Scale-Up Challenges: Process Engineering Constraints and Economic Barriers

The transition from high-titer laboratory constructs to economically viable industrial processes constitutes the most significant barrier in terpenoid biomanufacturing. Although metabolic engineering advancements have frequently led to impressive yields at the flask scale, these achievements often mask the substantial process engineering and economic challenges inherent in scaling up production [223]. This translational gap is characterized by distinct yet interconnected challenges across both microbial and plant-based production platforms. A critical and frequently underestimated challenge is the limitation in mass transfer. In large-scale bioreactors (exceeding 10,000 L), efficient oxygen transfer and nutrient mixing become technically challenging and energy-intensive [224]. This issue is particularly pronounced in plant cell and hairy root cultures, which exhibit heightened sensitivity to shear stress owing to their large cellular dimensions and rigid cell walls. Conventional high-shear impellers may induce cell lysis and compromise tissue integrity, thereby necessitating the implementation of specialized low-shear bioreactor configurations, such as airlift, wave, or bubble column systems [225]. Furthermore, heterogeneous culture conditions within large-scale bioreactors give rise to gradient zones in nutrient distribution, dissolved oxygen levels, and pH, leading to the emergence of cell subpopulations with variable productivity and potentially contributing to genetic instability over extended cultivation periods. In the context of in planta production, scaling generally involves expanding the agricultural footprint, which introduces numerous uncontrollable variables such as seasonal climatic fluctuations, pathogen outbreaks, and soil heterogeneity-factors that directly affect both the yield and consistency of terpenoid production. Finally, downstream processing (DSP) represents a major component of total production costs, particularly for intracellular terpenoids. The recovery and purification of low-abundance metabolites from complex biological matrices necessitate multi-step procedures that are not only expensive to scale but also generate substantial waste streams [223,226].

### 7.8. Regulatory and Societal Challenges

Gene-edited medicinal plants encounter a fragmented regulatory landscape, characterized by inconsistent regional regulatory approaches. In process-centric jurisdictions such as the European Union, stringent requirements are imposed under genetically modified organism (GMO) legislation. The European Food Safety Authority (EFSA) has acknowledged that existing regulatory guidelines are only “partially applicable” to Site-Directed Nuclease-1 (SDN-1) plants, thereby necessitating extensive trait-specific safety dossiers and detailed documentation of editing precision [227]. In regions such as the United States and Japan, product-focused regulatory frameworks leverage the non-transgenic characteristics of Site-Directed Nuclease-1 (SDN-1) modifications [227]. The European Food Safety Authority (EFSA) has confirmed that Site-Directed Nuclease-1 (SDN-1) modifications do not present any additional safety concerns when compared to conventionally bred varieties. This finding enables regulatory exemptions for gene edits that do not incorporate foreign DNA, provided that the introduced traits could also occur naturally. These regulatory provisions facilitated the 2021 commercialization of CRISPR-edited Sicilian Rouge high-GABA tomatoes, recognized as the world’s first genome-edited food product. In 2023, a rapid approval was granted for waxy corn, issued under the oversight of the Ministry of Agriculture, Forestry and Fisheries (MAFF) [228]. These successes demonstrate how societal acceptance can accelerate the commercialization of products within product-focused regulatory systems. This momentum is driven by transparent labeling practices and consumer education initiatives, which collectively contribute to mitigating the persistent “pharma-crop” stigma associated with medicinal applications.

In conclusion, while the field of terpenoid metabolic engineering has achieved remarkable advancements, significant bottlenecks remain. Among the challenges discussed, those most difficult to overcome are not purely technical in nature, but rather emerge at the critical interface between biology and process engineering. (i) the scale-up challenge: Arguably the most significant obstacle lies in the transition from high-titer laboratory-scale yields to economically viable industrial production. This involves limitations in mass transfer, the shear sensitivity of plant cells, heterogeneous bioreactor environments, and the prohibitively high costs associated with downstream processing. Addressing this challenge necessitates a holistic bioprocessing strategy, which is frequently neglected in early-stage research. (ii) the fundamental knowledge gap: Despite significant progress, our current understanding of complex pathway regulation, transport mechanisms, and the complete metabolic network remains incomplete, constituting a deeply entrenched bottleneck. The inability to accurately predict system-wide responses to metabolic engineering interventions frequently results in suboptimal yields and unintended trade-offs, such as growth penalties. Therefore, the identification of unknown transporters and the attainment of a quantitative, systems-level understanding of flux control are of critical importance. (iii) the regulatory and societal barrier: For plant-based production systems, navigating the fragmented and continuously evolving global regulatory landscape governing genetically modified organisms (GMOs) represents a non-technical yet critical bottleneck. Securing societal acceptance and regulatory approval for engineered medicinal plants involves a lengthy and uncertain process that can significantly impede commercialization. While technical challenges such as enzyme optimization and genetic transformation are being progressively addressed through advances in protein engineering and innovative tools like CRISPR, the challenges of scale-up, systems-level prediction, and regulatory compliance constitute broader, interconnected issues. These require coordinated multidisciplinary efforts that extend beyond the scope of metabolic engineering alone. Future progress will depend on the early integration of bioprocess engineering and techno-economic analysis into the design phase, complemented by sustained fundamental research aimed at deciphering biological complexity.

## 8. Prospects and Frontier Directions

**Figure 4:**
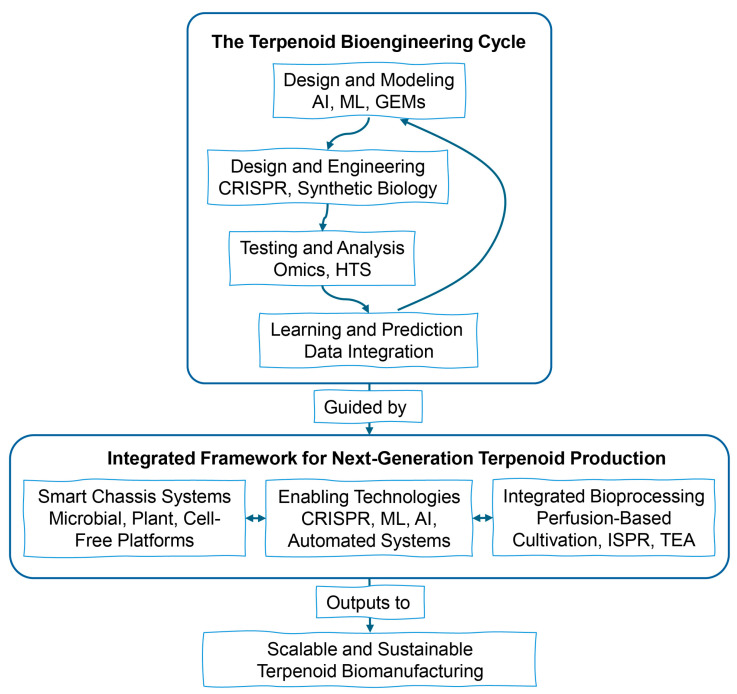
A comprehensive and integrated roadmap for advancing terpenoid biomanufacturing. Abbreviations: AI, artificial intelligence; ML, machine learning; GEMs, genome-scale metabolic models; CRISPR, clustered regularly interspaced short palindromic repeats; HTS, high-throughput screening; ISPR, in situ product removal; TEA, techno-economic analysis.

This section outlines pioneering strategies and multidisciplinary approaches that are expected to shape the next frontier of terpenoid metabolic engineering. Going beyond incremental improvements, it explores the integration of advanced bioprocessing, systems and synthetic biology, state-of-the-art gene editing technologies, and artificial intelligence to establish a novel paradigm for the design and production of terpenoids (Figure 4). The interconnections among these future directions are illustrated in Figure 4, which presents a comprehensive framework for next-generation terpenoid biomanufacturing. The following sections elaborate on the specific opportunities and challenges associated with integrated bioprocessing, systems biology, gene editing, synthetic biology, chassis development, and computational tools, providing a strategic roadmap to overcome existing bottlenecks and enable scalable, cost-effective, and sustainable production of high-value terpenoids.

### 8.1. Integrated Bioprocessing Strategies for Industrial Application and Scale-Up

Addressing the interconnected challenges of mass transfer limitations, shear sensitivity of plant cells, and the economic feasibility of downstream processing requires a holistic and integrated approach from the earliest stages of strain and process development. (i) the adoption of perfusion systems in plant cell cultures enables the continuous removal of spent media and toxic metabolites while retaining viable cells, thereby maintaining a productive environment over extended cultivation periods [229]. The integration of advanced process analytics with machine learning algorithms enables real-time monitoring and dynamic control of critical process parameters, facilitating the transition from empirical to predictive process management [173]. (ii) strategies such as in situ product removal (ISPR) play a critical role in mitigating feedback inhibition and cytotoxicity [230]. The in situ extraction of taxadiene has achieved recovery rates as high as 97% in a solid-phase system [231]. Furthermore, co-culture systems demonstrate significant potential as a biological strategy for enhancing terpenoid production. The co-cultivation of *Taxus* suspension cells with the endophytic fungus *Fusarium mairei* in a dual-bioreactor system resulted in a 38-fold increase in paclitaxel titer, rising from 0.68 mg/L in the uncoupled culture to 25.63 mg/L in the co-culture configuration [188]. (iii) techno-economic analysis (TEA) serves as a strategic design tool. By modeling production costs at commercial scale, TEA enables the identification of the most significant cost drivers [232]. In microbial terpenoid production, key cost drivers frequently encompass feedstock concentration, aeration rates, and, most importantly, the final product yield [232]. The explicit objective of such analyses is to provide clear and quantitative targets for researchers, thereby directing metabolic engineering efforts toward the most impactful improvements [232].

### 8.2. Deep Integration of Multi-Omics and Systems Biology for Predictive Modeling

The integration of multi-omics data (genomics, transcriptomics, proteomics, metabolomics, epigenomics) with genome-scale metabolic models (GEMs) and machine learning enables unprecedented precision in predicting metabolic flux bottlenecks, identifying regulatory targets, and optimizing engineering strategies [233]. Single-cell multi-omics resolves cell-type-specific metabolic networks, as exemplified in medicinal plants: scRNA-seq in *Gossypium* glandular trichomes revealed transcriptional hierarchies regulating gossypol biosynthesis and enabled CRISPR-mediated activation of *ERF12*, resulting in a 140% increase in yield [234]. Deep learning classification models demonstrate high accuracy in pathway prediction. Spec2Class achieves 73% top-1 accuracy for secondary metabolite classification based on chemical structures. Furthermore, GTC establishes benchmarks for multi-label pathway inference [235]. However, quantitative regression models for predicting metabolite concentrations remain underdeveloped due to the scarcity of temporal data and the absence of standardized benchmarks [235].

### 8.3. Advances in Gene Editing Technologies

CRISPR-based tools, including base editing (BE), prime editing (PE), multiplex editing, and transient ribonucleoprotein (RNP) delivery, are increasingly applied in plant biotechnology. These advancements improve the precision and safety of genome editing in plants. Base editing enables C-to-T and A-to-G substitutions without inducing double-strand breaks (DSBs), achieving an editing efficiency of 2.7–13.3% in rice [236]. Prime editing (PE) further enables all 12 base substitutions and small insertions or deletions (indels) without the need for donor templates, achieving regeneration frequencies of up to 21.8% in rice and wheat [236]. These technologies overcome the limitations imposed by low homologous recombination efficiency in recalcitrant species. For complex pathway engineering, multiplex editing enables concurrent targeting of rate-limiting terpenoid genes in *N. benthamiana*, thereby redirecting metabolic flux through the knockout of competing pathways [237]. The integration of CRISPR-based tools with metabolic engineering is progressing toward high-throughput multiplexing. Systems such as CRISPR-AID facilitate combinatorial metabolic engineering, enabling the simultaneous optimization of multiple genetic targets to rapidly identify superior production strains [84]. CRISPR-Cas9-assisted random mutagenesis resulted in a 10.5-fold increase in β-carotene production [84]. Critically, DNA-free ribonucleoprotein (RNP) delivery eliminates the risk of foreign DNA integration, thereby aligning with regulatory compliance. Transient RNP-mediated knockout of *CYP71D* genes in *N. benthamiana* enhances sesquiterpenoid yields [238], while USDA approvals for CRISPR-edited crops highlight their commercial viability [239]. However, the generation of heritable edits in metabolic pathways remains challenging, as somatic edits predominantly occur in the T1 generation, and stable inheritance typically requires T3 lineages [238].

### 8.4. Synthetic Biology and Modular Design Approaches

Synthetic biology applies engineering principles such as standardization, modularity, and orthogonality to deconstruct complex terpenoid biosynthetic pathways into interoperable genetic modules. This strategy enhances predictability and scalability. Although the foundational BioBrick standard inspired this approach, its direct implementation in terpenoid pathways remains limited. In contrast, BioBrick-inspired modular design enables pathway segmentation and optimization, as exemplified by the partitioning of the taxadiene pathway in *E. coli* into two independently tunable modules, resulting in a 15,000-fold increase in titer (~1 g/L) [17]. Orthogonal systems further ensure stable expression by decoupling engineered pathways from host regulatory mechanisms, as demonstrated by orthogonal T7 polymerases and riboswitches, which increased limonene yields in yeast and monoterpene titers in *E. coli* by 2-fold and 3- to 11-fold, respectively [25]. Dynamic regulatory circuits integrate metabolite-sensing feedback mechanisms, such as optogenetic controls or quorum-sensing systems, to automatically adjust gene expression in response to cellular conditions. This regulation balances precursor flux and mitigates toxicity, resulting in a 40% increase in amorphadiene production in yeast [25]. These strategies converge within plug-and-play platforms such as engineered *Yarrowia lipolytica* strains, which utilize standardized genetic modules to achieve 100-fold higher limonene and 8.4-fold increased valencene yields [240]. Collectively, modular design, orthogonal components, and intelligent regulatory systems transform terpenoid biosynthesis from an artisanal, trial-and-error approach into a predictable and systematic biomanufacturing process, although broader standardization remains a future objective [25].

### 8.5. Enzyme Engineering and Directed Evolutionary Strategies

Enzyme engineering and directed evolution are critical for enhancing the catalytic efficiency, specificity, and stability of terpenoid biosynthetic enzymes. This is especially relevant for cytochrome P450 monooxygenases (CYPs), terpene synthases (TPSs), and UDP-glycosyltransferases (UGTs). Rational design utilizes cryo-electron microscopy to elucidate dynamic conformational changes, integrated with molecular dynamics (MD) simulations for precise residue targeting, as illustrated by MD-guided thermostabilization strategies [241]. Directed evolution accelerates enzyme optimization through high-throughput screening innovations, such as microfluidic platforms and fluorescence-based reporter systems for CYP activity [241]. These approaches enable: (i) expansion of substrate scope Directed evolution of P450BM3 to access non-native terpenoids [242]; (ii) stability enhancement: Surface charge optimization in plant P450s improves heterologous expression in tobacco for taxol biosynthesis [243](Zhang et al., 2023); (iii) regioselectivity control: MD-simulated residue swaps in UGTs increasing glycosyla-tion efficiency by 2.5-fold [241]. Recent advances provide insights into electric field effects on P450 enzyme dynamics [244]. Collectively, these engineered enzymes enable novel terpenoid synthesis and yield enhancements while overcoming key challenges such as plant P450 solubility and host-environment tolerance.

### 8.6. Subcellular Compartment Engineering

Chloroplasts and mitochondria offer specialized compartments for the engineering of terpenoid biosynthesis, capitalizing on their metabolic compartmentalization and semi-autonomous genomic features. Chloroplast engineering utilizes high genome copy numbers (~10,000 per cell), prokaryotic-like expression systems, and endogenous terpenoid precursors (IPP/DMAPP) [245]. Chloroplast transformation facilitates high-level protein accumulation [246]; however, quantified terpenoid yields remain unreported despite pathway engineering in *Chlamydomonas reinhardtii* [247]. Critically, transporter engineering for the translocation of IPP/DMAPP remains unresolved, as only bicarbonate transporters have been successfully engineered to date [248]. Mitochondrial engineering focuses on enhancing acetyl-CoA flux. In yeast, pyruvate dehydrogenase (PDH) overexpression increased acetyl-CoA levels threefold, enabling compartmentalized terpenoid synthesis [249]. This resulted in quantifiable improvements: a 3.7-fold increase in α-santalene titers compared to cytosolic expression, 427 mg/L of amorpha-4,11-diene achieved through mitochondrion-localized pathways, and a 6-fold increase in geraniol production [249].

### 8.7. Development of an Efficient Universal Host System

Efficient plant chassis development prioritizes species exhibiting rapid growth kinetics, high biomass yield, well-characterized genomes, and amenability to genetic transformation. Specialized chassis leverage traditional medicinal plants; for instance, *Salvia miltiorrhiza* has been engineered through CRISPR-mediated knockout of *SmCPS1* to enhance tanshinone yields by eliminating competing terpenoid pathways, achieving 2.7 mg/g DW in hairy root systems [250]. *Perilla frutescens* has also demonstrated successful application of multiplex CRISPR editing, wherein the suppression of *FAD3* resulted in a 60% increase in therapeutic perillaldehyde [251](Verma et al., 2023). Universal chassis systems primarily utilize *N. benthamiana*, in which transient expression takes advantage of rapid biomass accumulation and efficient agroinfiltration [252]. Metabolic competition is actively minimized through VIGS-mediated *PSY* silencing, which doubled taxadiene titers to 48 μg/g DW; precursor pool expansion, elevating linalool fivefold; and multigene stacking, resulting in the production of artemisinin precursors at 130 mg/kg FW [253]. Although genome minimization in plants remains underdeveloped, targeted pathway disruption and transient engineering have established *N. benthamiana* as the premier universal platform for the production of therapeutic terpenoids. Future efforts should focus on CRISPR-based pathway pruning and flux modeling to systematically reduce endogenous metabolic competition [195].

### 8.8. Cell-Free Synthetic Biology Systems

Cell-free synthetic biology utilizes plant cell extracts or reconstituted purified enzyme systems to synthesize complex terpenoids in a precisely controlled in vitro environment. This approach overcomes cellular limitations, such as cytotoxicity, transport barriers, and regulatory complexity, while enabling rapid pathway prototyping more than 100 times faster than in vivo processes, facilitating simplified product separation, and allowing the synthesis of compounds that are toxic to living cells. Key advancements demonstrate the field’s maturity: orthogonal cofactor regeneration achieved a 65% yield in nepetalactol synthesis and reduced cofactor costs by more than 50-fold through NAD(P)H recycling [254]. Engineered yeast systems produced 2.23 g/L of limonene, while optimized platforms maintain productivities exceeding 100 mg/L/h at scales exceeding 100 L [254]. Enzyme stabilization through nanochannel confinement maintained activity for over 1740 reaction cycles, or 72 h, effectively addressing long-term instability [254]. However, challenges persist in cofactor economics, as NADPH regeneration costs exceed $1000 per kilogram in the absence of recycling; pathway scalability, with a fivefold increase in shunt products at the 10-mL scale compared to 200 μL; and enzyme sourcing, with fewer than 10% of plant terpenoid pathways reconstituted [254]. Despite these challenges, the system demonstrates exceptional capability in synthesizing pharmaceutically relevant terpenoids, such as triterpenoid betulinic acid with a productivity of 18.7%, positioning it for industrial adoption in the manufacturing of high-value compounds [254]. Beyond cell-free systems, the next frontier in spatial organization may involve the de novo engineering of membrane-less organelles within living cells through liquid–liquid phase separation (LLPS), thereby creating dedicated nano-bioreactors for terpenoid synthesis [84].

### 8.9. Integration of Artificial Intelligence and Machine Learning

Artificial intelligence (AI) and machine learning (ML) are profoundly transforming metabolic engineering, particularly in the field of terpenoid synthesis. In target prediction, deep learning models leverage databases such as KEGG and MetaCyc to identify novel genes involved in terpenoid biosynthesis and to discover enzymatic regulators [255]. For pathway design, AI-driven platforms such as BioNavI-NP and novoStoic enable the de novo construction of mass-balanced biosynthetic routes; transfer learning strategies further improve top-10 pathway prediction accuracy by approximately 20% through pretraining on chemical synthesis databases [256]. In enzyme engineering, artificial intelligence significantly reduces the burdens associated with experimental screening. Tools such as MutaCYP achieve 84.6% accuracy in classifying functional mutations in cytochrome P450s. Additionally, DeepP450 attains AUROC values ranging from 0.89 to 0.98 across major CYP subfamilies [173]. For bioprocess control, machine learning models optimize nutrient and hormone parameters; however, peer-reviewed evidence directly linking ML-optimized light spectra to terpenoid yields remains limited [167].

### 8.10. Focus on Non-Model Medicinal Plants

Most plants with documented medicinal value are classified as “non-model species,” characterized by the absence of reference genomes and limited transformation protocols. However, advances in high-throughput sequencing now enable the genomic characterization of these species: RNA-seq facilitates gene discovery in *Gentiana rigescens* and *Phyllanthus amarus* in the absence of reference genomes, while chromosome-scale assemblies reveal terpenoid biosynthetic diversity [257]. Concurrently, transient CRISPR-Cas9 techniques partially overcome transformation barriers, as demonstrated by PEG-mediated RNP delivery achieving 71.07% editing efficiency in *Salvia miltiorrhiza* hairy roots for the engineering of tanshinone biosynthesis [258]. Despite these advances, critical gaps persist in the research. No peer-reviewed studies have confirmed the application of transient CRISPR techniques in rare traditional Chinese medicinal herbs, and novel terpenoid structures remain unlinked to genomic validations.

### 8.11. End-to-End Integration and Collaborative Innovation

Future success in medicinal plant terpenoid research requires comprehensive integration spanning from fundamental discovery to commercialization. Basic research must focus on elucidating the genomic architecture and metabolic networks. Studies have demonstrated that transcription factors and epigenetic mechanisms, such as DNA demethylation, contribute to enhanced tanshinone accumulation in *Salvia miltiorrhiza* [259]. Concurrently, metabolic engineering necessitates the synergistic application of advanced tools: CRISPR-based editing of *Taxus spp*. for taxadiene overproduction and synthetic biology platforms that reconstruct terpenoid pathways in yeast [5,16]. Downstream integration necessitates green extraction technologies and in situ modifications, such as engineered glycosyltransferases that improve terpenoid solubility in microbial chassis systems [26]. Industrial translation crucially relies on academia-industry partnerships, as demonstrated by the scale-up of a semi-synthetic taxol precursor. Such collaborations effectively address scale-up economics and regulatory challenges, thereby accelerating patient access to plant-derived therapeutics [23].

## 9. Conclusions

This review highlights metabolic engineering as a critical strategy for the sustainable production of terpenoids. It emphasizes several key advancements in the field. One significant development is the utilization of genomic insights; studies have demonstrated that chromosome-level genomes and single-cell omics offer a comprehensive understanding of cell-type-specific regulatory mechanisms. Research on glandular trichome-enriched artemisinin biosynthesis genes has illustrated the potential for precise manipulation of biosynthetic pathways. Furthermore, engineering breakthroughs have significantly contributed to enhanced production efficiency. Evidence suggests that CRISPR-based tools, such as *SmABCG1* knockout, can improve tanshinone export. Additionally, enzyme optimizations enabled by machine learning have resulted in substantial yield improvements, with reports documenting a 200-fold increase in UGT73P12 catalytic efficiency. Collectively, these advancements have conclusively demonstrated the feasibility of heterologous reconstruction of complex terpenoid pathways in optimized chassis systems, including yeast and *N. benthamiana*. This progress has transitioned the production of several high-value terpenoids from theoretical concepts to experimentally validated realities, bringing them significantly closer to commercial viability. The field has now progressed beyond proof-of-concept studies and is addressing the next critical frontier: scaling up production while ensuring economic sustainability. Pioneering studies have established foundational protocols for scaled bioreactor cultivation and microbial production, offering a crucial framework for future industrial translation. The primary challenges are no longer exclusively biological but increasingly require the integration of engineering solutions to overcome limitations in mass transfer and downstream processing costs. However, several challenges, such as genetic instability and regulatory concerns, remain to be addressed; policies governing SDN-1 plants continue to pose significant barriers. Looking ahead, the integration of AI-driven metabolic modeling, cell-free production systems, and photoautotrophic chassis organisms is anticipated to bridge the gap between laboratory-scale innovations and commercial-scale applications. Realizing the full potential of plant-based “green factories” for the production of high-value terpenoids will require collaborative efforts that span fundamental research, synthetic biology, and industrial implementation.

## Figures and Tables

**Table 1:** Comparative analysis of the major platforms for terpenoid bioproduction.

Aspect	Native Medicinal Plants	Microbial Chassis	Heterologous Plant Hosts
Key Advantages	Native enzymatic context for complex modifications;	Rapid growth & high cell density; Well-established genetic tools & high-throughput screening; Scalable fermentation	Eukaryotic PTMs and compartmentalization; Low-cost biomass production (agroinfiltration); Capable of complex pathways
	Pre-existing storage structures		
Major Limitations	Long growth cycles;	Cytotoxicity of intermediates; Lack of specific P450s/UGTs; Cofactor balancing issues; High substrate costs	Transient expression limitations; Metabolic competition with endogenous pathways; Scale-up challenges for extraction
	Low yields;		
	Complex genetics & recalcitrance to transformation;		
	Ecological concerns		
Max. Yields	Artemisinin: ~1.2% DW [14]; Paclitaxel: ~0.05% DW [15]	Artemisinic acid: >25 g/L (yeast) [16]; Taxadiene: >1 g/L (*E. coli*) [17]; Protopanaxadiol: 11 g/L (yeast) [9] Ginsenoside K: 5.74 g/L (yeast) [18]	Taxadiene: ~48 µg/g DW (chloroplast-targeted) [19]; Triterpenes: 37.9 mg/g DW [20]
Cost & Scalability	High agricultural land & labor cost;	Fermentation costs significant but controllable; Highly scalable to industrial bioreactors (10,000+ L)	Medium cost; Scaling requires large greenhouse space, not yet industrial
	Difficult to scale, season-dependent		
Tech. Maturity (TRL)	Medium	High	Medium-High
Ideal Terpenoid Targets	High-value compounds already produced by the plant;	Volatile mono/sesquiterpenes; Triterpene scaffolds; Non-natural derivatives via combinatorial biosynthesis	Complex diterpenes/triterpenes; Molecules requiring plant-specific P450s/UGTs; Rapid prototyping of pathways
	Molecules requiring extensive, plant-specific modifications		

Abbreviations: DW: Dry Weight; PTMs: Post-Translational Modifications; TRL: Technology Readiness Level.

**Table 2:** Representative applications of subcellular targeting in terpenoid metabolic engineering.

Target Compound	Host	Target Organelle	Engineering Strategy	Key Targeting Signal	Outcome	Reference
Taxadiene	*N. tabacum*	Chloroplast	Plastid-targeted expression of Taxus taxadiene synthase	Chloroplast transit peptide	5.6 μg/g DW	[64,95]
Valencene	*S. cerevisiae*	Mitochondria	Mitochondrial-targeted valencene synthase	COX4 MTS	3-fold increase	[41,104]
Triterpenoids	*N. tabacum*	Chloroplast	Reconstitution of cytosolic MVA pathway in chloroplasts	Plastid-targeted HMGR, FPS	Significant yield enhancement	[30,105]
Artemisinic acid	*S. cerevisiae*	Endoplasmic Reticulum (ER)	ER-membrane anchoring of CYP71AV1 and CPR	Cytochrome P450 N-terminal anchor	Improved electron transfer, higher oxidation efficiency	[16]
limonene	*S. cerevisiae*	Cytoplasm	Orthogonal pathway (*SlNDPS1* + *LS*) + *ERG20* repression by *HXT1* promoter	N/A (cytosolic expression)	917.7 mg/L (6-fold increase)	[106]

Abbreviations: DW, dry weight; COX4, cyclooxygenase-4; MTS, mitochondrial targeting sequence; HMGR, 3-hydroxy-3-methylglutaryl-CoA reductase; FPS, farnesyl diphosphate synthase; CPR, cytochrome P450 reductase; SlNDPS1, *Solanum lycopersicum* neryl diphosphate synthase 1; LS, limonene synthase; ERG20, ergosterol biosynthesis gene 20; HXT1, hexose transporter 1.

## Abbreviations

The following abbreviations are used in this manuscript:

ABC	ATP-Binding Cassette
ADS	Amorpha-4,11-Diene Synthase
AI	Artificial Intelligence
ALDH1	Aldehyde Dehydrogenase 1
ATAC-seq	Assay for Transposase-Accessible Chromatin with high-throughput sequencing
ATP	Adenosine Triphosphate
BE	Base Editing
ChIP-seq	Chromatin Immunoprecipitation sequencing
COX-2	Cyclooxygenase-2
CPR	Cytochrome P450 Reductase
CRISPR	Clustered Regularly Interspaced Short Palindromic Repeats
CRISPR-Cas9	CRISPR-associated protein 9
CRISPRi	CRISPR interference
CYP	Cytochrome P450
DMAPP	Dimethylallyl Diphosphate
DXS	1-Deoxy-D-Xylulose-5-Phosphate Synthase
EFSA	European Food Safety Authority
ER	Endoplasmic Reticulum
FAD3	Fatty Acid Desaturase 3
FPP	Farnesyl Diphosphate
G6PDH	Glucose-6-Phosphate Dehydrogenase
GA3	Gibberellin A3
GC-MS	Gas Chromatography-Mass Spectrometry
GEMs	Genome-scale Metabolic Models
GGPP	Geranylgeranyl Diphosphate
GMO	Genetically Modified Organism
GPP	Geranyl Diphosphate
GRNs	Gene Regulatory Networks
HMG-CoA	3-Hydroxy-3-Methylglutaryl-CoA
HMGR	3-Hydroxy-3-Methylglutaryl-CoA Reductase
HRMS	High-Resolution Mass Spectrometry
IDI	Isopentenyl Diphosphate Isomerase
IMS	Ion Mobility Spectrometry
IPK	Isopentenyl Phosphate Kinase
IPP	Isopentenyl Diphosphate
iNOS	Inducible Nitric Oxide Synthase
IUP	Isopentenol Utilization Pathway
JA	Jasmonate
JAZ	Jasmonate ZIM-domain
LC-MS/MS	Liquid Chromatography-Tandem Mass Spectrometry
MAFF	Ministry of Agriculture, Forestry and Fisheries (Japan)
MD	Molecular Dynamics
MeJA	Methyl Jasmonate
MEP	Methylerythritol Phosphate pathway
ML	Machine Learning
MVA	Mevalonate pathway
NADPH	Nicotinamide Adenine Dinucleotide Phosphate (reduced form)
NF-κB	Nuclear Factor kappa B
NMR	Nuclear Magnetic Resonance
OptoAMP	Optogenetic Amplification
oxPPP	Oxidative Pentose Phosphate Pathway
PAL	Phenylalanine Ammonia-Lyase
PDH	Pyruvate Dehydrogenase
PE	Prime Editing
PEPC	Phosphoenolpyruvate Carboxylase
POR	Protochlorophyllide Oxidoreductase
PSY	Phytoene Synthase
PTMs	Post-Translational Modifications
RNAi	RNA Interference
RNA-seq	RNA Sequencing
RNP	Ribonucleoprotein
ROS	Reactive Oxygen Species
SA	Salicylic Acid
scRNA-seq	Single-cell RNA Sequencing
SDN-1	Site-Directed Nuclease 1
SEM	Structural Equation Modeling
SQS	Squalene Synthase
SRM	Selected Reaction Monitoring
SWATH-MS	Sequential Window Acquisition of All Theoretical Mass Spectra
TF	Transcription Factor
TLA	Three Letter Acronym (included as per example)
TMT	Tandem Mass Tag
TPS	Terpene Synthase
UGT	UDP-Glycosyltransferase
USDA	United States Department of Agriculture
VIGS	Virus-Induced Gene Silencing
WGCNA	Weighted Gene Co-expression Network Analysis
